# Lower temperatures reduce type I interferon activity and promote alphaviral arthritis

**DOI:** 10.1371/journal.ppat.1006788

**Published:** 2017-12-27

**Authors:** Natalie A. Prow, Bing Tang, Joy Gardner, Thuy T. Le, Adam Taylor, Yee S. Poo, Eri Nakayama, Thiago D. C. Hirata, Helder I. Nakaya, Andrii Slonchak, Pamela Mukhopadhyay, Suresh Mahalingam, Wayne A. Schroder, William Klimstra, Andreas Suhrbier

**Affiliations:** 1 QIMR Berghofer Medical Research Institute, Brisbane, Queensland, Australia; 2 Australian Infectious Disease Research Centre, Brisbane, Queensland, Australia; 3 Institute for Glycomics, Griffith University, Gold Coast, Queensland, Australia; 4 Department of Virology I, National Institute of Infectious Diseases, Tokyo, Japan; 5 School of Pharmaceutical Sciences, University of Sao Paulo, Sao Paulo, Brazil; 6 School of Chemistry and Molecular Biosciences, University of Queensland, Brisbane, Queensland, Australia; 7 Department of Microbiology and Molecular Genetics Center for Vaccine Research University of Pittsburgh, Pittsburgh, Pennsylvania, United States of America; Purdue University, UNITED STATES

## Abstract

Chikungunya virus (CHIKV) belongs to a group of mosquito-borne alphaviruses associated with acute and chronic arthropathy, with peripheral and limb joints most commonly affected. Using a mouse model of CHIKV infection and arthritic disease, we show that CHIKV replication and the ensuing foot arthropathy were dramatically reduced when mice were housed at 30°C, rather than the conventional 22°C. The effect was not associated with a detectable fever, but was dependent on type I interferon responses. Bioinformatics analyses of RNA-Seq data after injection of poly(I:C)/jetPEI suggested the unfolded protein response and certain type I interferon responses are promoted when feet are slightly warmer. The ambient temperature thus appears able profoundly to effect anti-viral activity in the periphery, with clear consequences for alphaviral replication and the ensuing arthropathy. These observations may provide an explanation for why alphaviral arthropathies are largely restricted to joints of the limbs and the extremities.

## Author summary

Chikungunya virus (CHIKV) and Ross River virus are mosquito-borne alphaviruses that cause epidemics of human arthritic disease that usually last from weeks to months. Arthropathy predominantly manifests in the joints of limbs and the joints at the extremities (e.g. hand and feet). Herein we show a surprisingly large reduction in viral loads and foot arthropathy in mice when animals were housed at 30°C rather than the conventional 22°C, with the feet of the former mice being ≈3–4°C warmer. Using RNA-Seq analyses of mice feet, we illustrate that this small increase in temperature results in a significant increase in both the unfolded protein response and anti-viral type I interferon responses. Taken together these results suggest that the predominantly peripheral alphaviral arthropathy is due to the usually slightly lower temperature of limbs and extremities, which results in less effective type I interferon responses and a subsequent increase in viral loads and ensuing arthritic disease.

## Introduction

Studying the role of temperature in regulating viral infections and viral pathologies has an eclectic history. The Australian nurse, Elizabeth Kenny (1880–1952), pioneered the treatment of polio with application of heat packs to affected limbs [1,2], although the mechanistic basis for this treatment has not been identified [3]. A number of reasons for the increase in respiratory tract infections during the winter months have been proposed, with *in vitro* experiments in cell lines suggesting a role for improved type I interferon (IFN) responses at 37°C compared with lower temperatures [4–6]. A range of other factors have also been implicated including cold stress, respiratory tract vasoconstriction, humidity, transmission, and host behavior [7,8]. Fever has also been viewed as potentially beneficial in fighting viral infections [9,10], although effects may be quite marginal [11,12]. Mice housed at 30°C have been shown to have increased CD8 T cell activity compared with mice held at 20–26°C, although this study was concerned with anti-cancer responses [13]. Elevated ambient temperatures have been shown to increase expression of type I IFN stimulated genes (ISGs), TNF and IL-1, and protect Japanese flounders against Hirame rhabdovirus [14]. Olive flounders infected with hemorrhagic septicemia virus if held at 20°C rather than 15°C also showed improved survival, increased virus-induced apoptosis [15], and increased levels of Toll-like receptor 7 and Interferon Response Factor 7 (IRF7) [16]. In mosquitoes, maintenance at lower temperatures favors replication of chikungunya virus (CHIKV) and yellow fever virus by suppressing the efficiency of the insect’s anti-viral ‘RNA-induced silencing complex’ [17].

A number of mosquito-borne viruses in the genus alphavirus (family Togaviridae) cause outbreaks of human rheumatic disease around the world; these include CHIKV, the primarily Australian Ross River virus (RRV) and Barmah Forest virus, the African o’nyong-nyong virus, the Sindbis group of viruses and the South American Mayaro virus [18]. CHIKV has recently re-emerged to produce the largest documented outbreak of CHIKV disease ever recorded; the outbreak began in 2004 in Africa, spread across Asia to Indonesia, and reached the Americas in 2013 [19–21]. Millions of CHIKV cases have been reported [22]. RRV causes about 4–5000 cases per annum in Australia, and in 1979–80 was responsible for a large epidemic in the South Pacific [23]. Symptomatic infection of adults with these alphaviruses is associated with acute and chronic (often debilitating) polyarthralgia and/or polyarthritis, which usually lasts weeks to months, occasionally longer [18]. Alphaviral arthritic disease is generally symmetrical with joints in limbs and at the extremities most commonly affected (e.g. feet, hands, ankles, wrists, knees, elbows) [23–26]. Arthritic inflammation arises from viral infection of joint and surrounding tissues with viral RNA and/or viral proteins stimulating innate and adaptive pro-inflammatory immune responses [18,27–31].

A number of mouse models for alphavirus infection and disease have been developed [32–38]. The adult wild-type C57BL/6 mouse model of CHIKV infection and disease used herein involves injection of CHIKV s.c. into feet, and recapitulates the viremia and arthropathy seen in humans [32]. RNA-Seq analyses also revealed a good concordance in inflammatory gene induction between the mouse model and CHIKV patients [27]. The latter analyses also illustrated the dominance of the type I IFN response, with ISGs representing ≈50% of CHIKV-induced genes [27]. A key role for the type I IFN response in controlling alphaviral infection is well established [38–40], with type I interferon receptor deficient (IFNAR^-/-^) mice rapidly succumbing to CHIKV infection [35,41]. The transcription factors, Interferon Response Factor 3 (IRF3) and IRF7 are critical for innate protection, with CHIKV-infected IRF3/7^-/-^ (double knockout) mice unable to produce detectable type I IFNs, resulting in high viremias, fever, hemorrhagic shock and mortality within a few days (a disease outcome only occasionally seen in humans) [41].

Herein we provide evidence that the reason for the largely peripheral alphaviral arthropathy seen in humans is that joints in the limbs and the extremities are a few degrees cooler than the core temperature of ≈37°C, with slightly cooler temperatures associated with suboptimal anti-viral type I IFN responses. Increasing the mouse housing temperature from 22°C to 30°C increased foot temperatures; this led to increased activation of the unfolded protein response (UPR) and improved anti-viral type I IFN responses in the feet. CHIKV replication was subsequently reduced and peripheral arthropathy was substantially ameliorated.

## Results

### Mice housed at 30°C show substantial reductions in viral loads and arthritic disease

**Fig 1:**
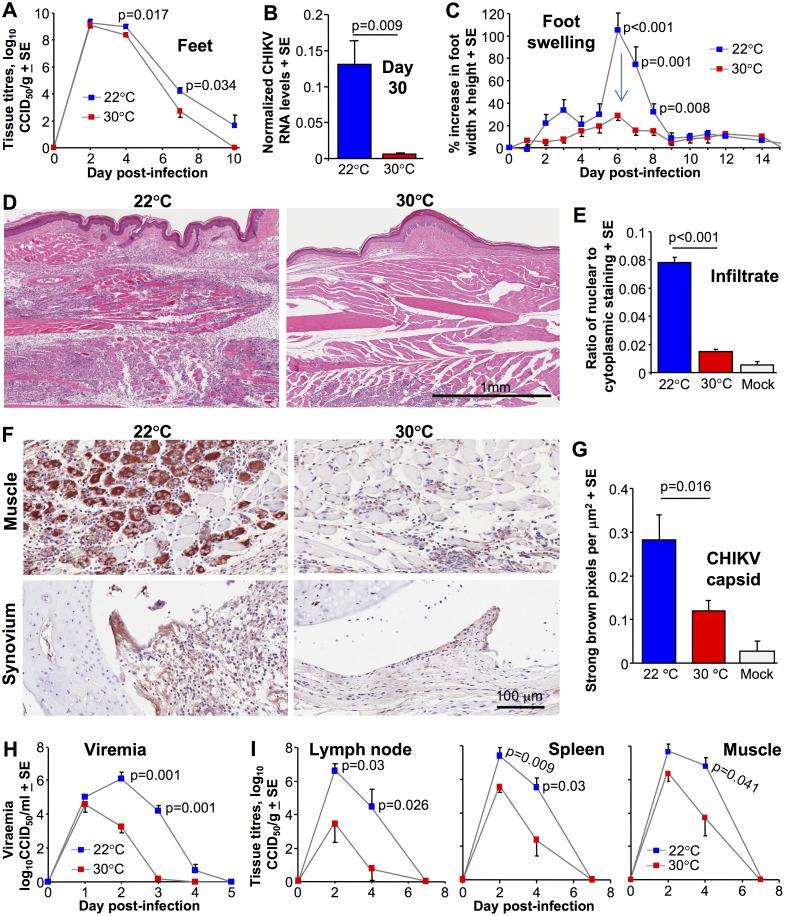
The effect of ambient temperature on CHIKV infection and disease. (A) CHIKV titers in feet of mice held at 30°C or 22°C after CHIKV inoculation s.c. into the feet. (Statistics by t test, day 4 or Kolmogorov-Smirnov test, day 7, n = 12/16 feet from 6/8 mice per group, data was obtained from two independent experiments). (B) Relative CHIKV RNA levels in feet on day 30 post infection. RNA levels normalized to RPL13A. (Statistics by Kolmogorov-Smirnov test, n = 6 feet from 6 mice per group). (C) Foot swelling for mice housed at 30°C or 22°C (Statistics by Kolmogorov-Smirnov or Mann-Whitney U tests, n = 12–16 feet from 6–8 mice per group, data was obtained from two independent experiments). (D) Examples of H&E staining of arthritic feet day 7 post infection for mice housed at 22°C and 30°C. At 22°C blue staining nuclei of infiltrating cells can be seen throughout the section. At 30°C blue staining nuclei of infiltrating cells are less abundant, with a patch evident bottom middle/left of the section. (E) Aperio pixel count quantification of the leukocyte infiltrates (exemplified in D), expressed as a ratio of nuclear to cytoplasmic staining ratios; (infiltrating leucocytes have relatively high nucleus to cytoplasm ratios [36]). N = 6 mice and feet per group, with 3 sections per foot used to produce a mean for each animal; statistics by t test. (F) Immunohistochemistry of muscle and synovial tissues (using an anti-CHIKV capsid monoclonal antibody) on day 7 post infection in feet of mice held at 22°C and 30°C. Staining of mock infected controls is shown in S1B Fig. (G) Aperio pixel count quantization of the immunohistochemistry (n = 5/6 feet from 5/6 mice for each temperature), with three sections per foot providing a mean for each foot. Statistics by Mann Whitney U test. (H) Viremia of mice housed at an ambient temperature of 30°C or 22°C and infected s.c. in the feet. (Statistics by Mann-Whitney U tests, n = 6–8 mice per group). (I) CHIKV titers in inguinal lymph nodes (two from each mouse pooled), spleen and quadriceps muscle (two from each mouse pooled) of mice held at 30°C or 22°C. (Statistics by Mann-Whitney U test, t test or Kolmogorov-Smirnov tests, n = 6–8 mice per group, data was obtained from two independent experiments).

Using the adult wild-type mouse model of CHIKV viremia and arthritis described previously [27,32,36,37], C57BL/6J female mice were inoculated subcutaneously (s.c.) into the feet with CHIKV (Reunion Island isolate). Mice were housed at an ambient temperature of 22 ± 1°C (as per standard animal house temperature) or 30 ± 1°C five days before viral inoculation and were kept at these temperatures until the end of the experiment. (Mice housed at the higher temperature did not show any overt changes in appearance or behavior). As described previously [32], high viral loads are reached in the feet within 2 days post inoculation (Fig 1A). Thereafter, the feet of mice housed at 30°C showed 1–2 log lower viral loads than feet of mice housed at 22°C (Fig 1A), with qRT PCR also showing lower CHIKV RNA levels (S1A Fig). In addition, CHIKV RNA levels in feet on day 30 post-infection were ≈20 fold lower in feet of mice kept at 30°C (Fig 1B). In this model for mice housed at 22°C, CHIKV RNA persists in feet for up to 100 days post infection [36], with persistent viral RNA associated with chronic arthritic disease [28,30,42,43].

Foot swelling, which is a measure of acute arthritis and peaks on day 6/7 in this model [27,32,36], was substantially and significantly reduced in mice kept at 30°C (Fig 1C, arrow). H&E staining of foot sections during acute arthritis illustrated the previously described [32] prodigious mononuclear inflammatory infiltrate in feet of CHIKV-infected mice housed at 22°C (Fig 1D, 22°C). In contrast, mice housed at 30°C showed substantially lower numbers of infiltrating cells (Fig 1D, 30°C). Quantification of the histology (by Aperio pixel count [27]) confirmed this difference was highly significant (Fig 1E). In addition, immunohistochemistry [44] showed less CHIKV antigen in foot tissues of mice kept at 30°C (Fig 1F; mock samples are shown in S1B Fig); with quantification demonstrating significance (Fig 1G).

Given (i) that CHIKV infections are effectively controlled by type I interferon (IFN) responses [27,38,39,41] and (ii) previous *in vitro* data suggests lower type I IFN production and activity at lower temperatures [4–6], these results suggest that for mice housed at 30°C, type I IFN responses in feet are improved, leading to reduced CHIKV replication and a subsequent decrease in viral arthropathy. Mice housed at 30°C also showed a substantially (≈2–4 log) and significantly lower viremia on days 2 and 3 post infection (Fig 1H), with virus tissue titers in lymph nodes, spleen and muscle similarly affected (Fig 1I). These results suggest an increase in systemic antiviral activity (see below).

### The temperature phenotype requires intact type I IFN responses

**Fig 2:**
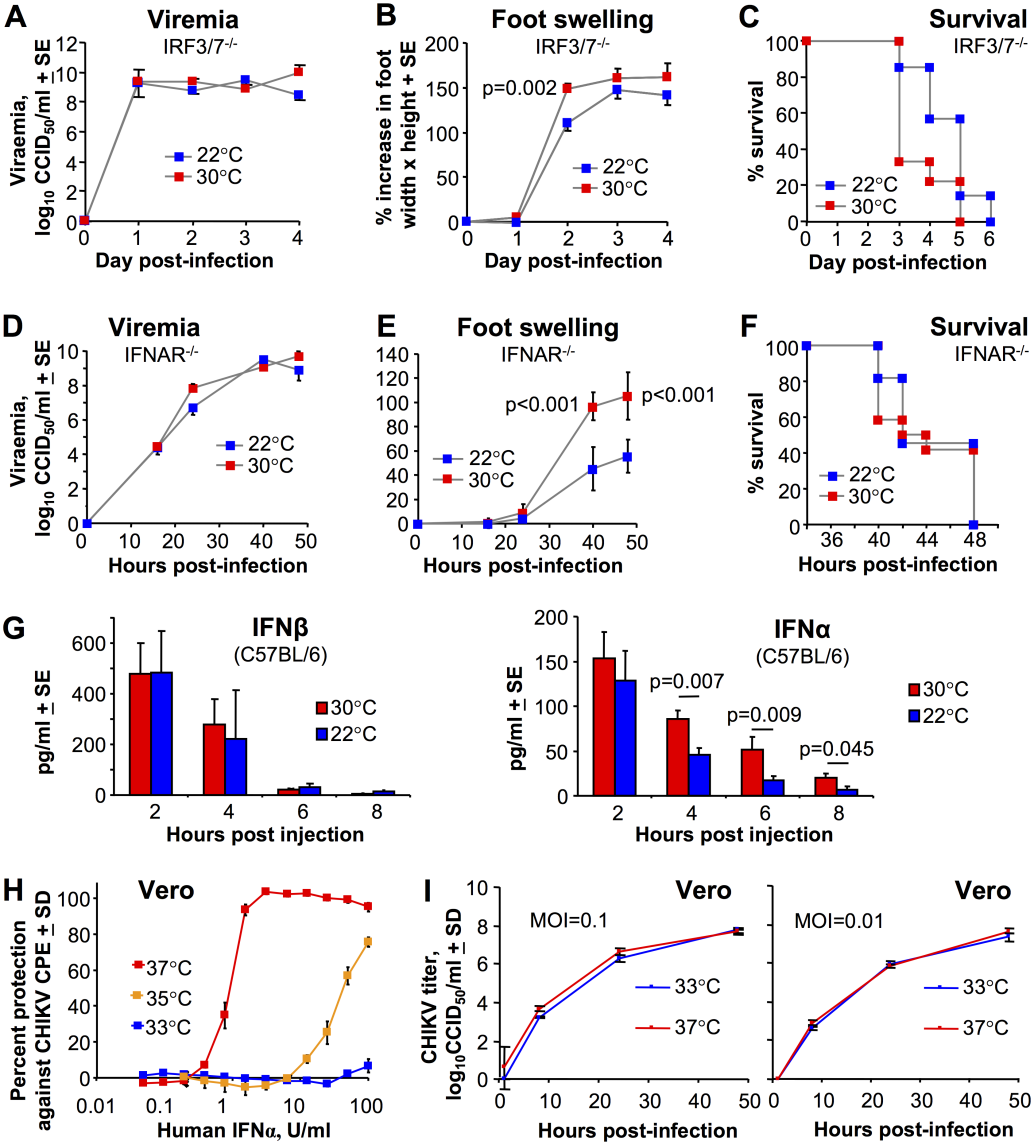
The role of the type I interferon response. (A) Viremia in IRF3/7^-/-^ mice housed at 30°C or 22°C after CHIKV infection s.c. into the feet (n = 9/12 mice per group). (B) Foot swelling in IRF3/7^-/-^ mice housed at 30°C or 22°C and infected s.c. in the feet. (Statistics by t test, n = 14–18 feet from 7–9 mice per group). (C) Survival of IRF3/7^-/-^ mice housed at 30°C or 22°C and infected s.c. in the feet. (Statistics by Mantel-Cox Log Rank test, p = 0.071, n = 7–9 mice per group). (Data for A-C was obtained from 2 independent experiments). (D) As for A except viremia in IFNAR^-/-^ mice (n = 5/6 mice per group). (E) As for B except foot swelling in IFNAR^-/-^ mice. (Statistics by t test, n = 12 feet from 6 mice per group). (F) As for C except survival of IFNAR^-/-^ mice (n = 11/12 animals per group; data obtained from 2 independent experiments). (G) Serum IFNβ and IFNα levels after s.c. injection of 5 μg poly(I:C)/jetPEI into feet. Statistics by t test except for 6 hours where a Mann Whitney U test was used due to non-normal data distribution (n = 6 mice). (H) Vero cell were incubated at the indicated temperatures and treated with IFNα for 4 hrs followed by infection with CHIKV (MOI = 0.05). Cytopathic effect (CPE) was measured after 3 days. (I) Vero cells at the indicated temperatures were infected with CHIKV at the indicated multiplicity of infection (MOI), were washed, and viral titers in the supernatants assessed at the indicated hours after washing. Data generated from 3 replicate wells.

To determine whether type I IFN responses are required for the reductions in viral loads seen for mice kept at 30°C (Fig 1A, 1B, 1H and 1I), IRF3/7^-/-^ were housed at 30°C or 22°C and were infected with CHIKV s.c. in the feet. IRF3/7^-/-^ mice do not generate detectable serum IFNα/β levels following CHIKV infection and usually die within 5–6 days [41]. In contrast to wild-type mice, neither the viremia (Fig 2A), nor the foot swelling (Fig 2B) was reduced in IRF3/7^-/-^ mice housed at 30°C relative to IRF3/7^-/-^ mice housed at 22°C. Foot swelling was actually slightly higher at 30°C (reaching significance on day 2) (Fig 2C). In addition, survival of CHIKV-infected IRF3/7^-/-^ mice was also marginally less for mice held at 30°C, although the difference only approached significance (p = 0.071). Thus IRF3/7^-/-^ mice, in contrast to C57BL/6 mice, did not show reduced viral replication and disease when housed at 30°C; if anything disease was slightly increased at this temperature in IRF3/7^-/-^ mice.

The above experiments were repeated in IFNAR^-/-^ mice; these mice are unable to respond to IFNα/β due to the lack of a functional IFNα/β receptor and die rapidly after CHIKV infection [35,41]. In contrast to wild-type mice (Fig 1H) (but similar to IFNAR^-/-^ mice, Fig 2A), there was no difference in viremia between IFNAR^-/-^ mice housed at 22°C and 30°C (Fig 2D). Foot swelling in IFNAR^-/-^ mice was significantly higher at 30°C (as in IRF3/7^-/-^ mice, Fig 2B); opposite to the reduced swelling seen in C57BL/6 mice housed at 30°C (Fig 1C). The reduced viremia (Fig 1H) and foot swelling (Fig 1C) seen at 30°C in wild-type mice was thus not recapitulated in either IRF3/7^-/-^ (Fig 2A and 2B) or IFNAR^-/-^ mice (Fig 2D and 2E). One should note that CHIKV-induced foot swelling in the latter two type I IFN response-deficient mice is quite distinct from that seen in C57BL/6 mice (Fig 1D), and is largely due to hemorrhage and shock-associated edema [41]. Lower temperatures are known to reduce edema [45]. There was no significant difference in the survival of CHIKV-infected IFNAR^-/-^ mice housed at 22°C or 30°C (Fig 2F).

The ability of the higher ambient temperature of 30°C to reduce CHIKV infection and arthritis in wild-type mice (Fig 1) thus requires the presence of key members of the type I IFN pathway.

### Increased serum IFNα levels in mice housed at 30°C

The data so far argues that for wild-type mice infected in the feet, type I IFN responses work better when animals are housed at 30°C. Fig 1H & 1I also suggest increased systemic type I IFN activity at 30°C. Injection of poly(I:C) induces very rapid and transient serum type I IFN levels [46]. Furthermore, injection of poly(I:C)/jetPEI into feet represents a model of dsRNA-induced arthritis, with jetPEI (a transfection reagent) helping to deliver poly(I:C) to the cytoplasm, mimicking cytoplasmic viral RNA replication and stimulating cytoplasmic RNA sensors [47]. Mice were housed at 22°C or 30°C and were injected s.c. in the feet with poly(I:C)/jetPEI, and IFNα and IFNβ levels were then measured in serum. IFNβ levels at all time points tested, and IFNα levels at 2 hours post injection, were not significantly different. However, at 4, 6 and 8 hours, IFNα levels were significantly 2–3 fold higher for mice housed at 30°C. IFNβ (and to some extent IFNα4) are generated in the first phase of the type I IFN response, with subsequent signaling via the IFNα/β receptor leading to up-regulation of *inter alia* IRF7 and the second phase of amplified production of IFNαs [41,48,49]. Increasing the temperature of feet thus appears to promote the secondary amplification loop, rather than the initial sensing of dsRNA and the production of IFNβ [49].

When CHIKV infection rather than poly(I:C)/jetPEI injection was tested, the reverse result was obtained, with serum IFNα levels (on day 2) higher in mice housed at 22°C than in mice housed at 30°C (S2A Fig). However, on day 2 the viremia in mice housed at 22°C was already ≈3 logs higher than in mice housed at 30°C (S2A Fig). Thus the two groups differed not only in terms of housing temperatures, but also vastly different viral loads. This complication is clearly avoided by using poly(I:C)/jetPEI rather than CHIKV infection (see below). Nevertheless, Ingenuity Pathway Analysis (IPA) of differentially expressed genes (DEGs) obtained from RNA-Seq after CHIKV infection also provided some evidence that the secondary type I IFN amplification loop is favored at 30°C (S2B Fig).

### Substantially reduced anti-CHIKV activity of IFNα at lower temperatures

To further investigate the effects of temperature on IFNα activity, Vero cells which can respond to, but cannot make IFNα/β, were cultured at 37°C, 35°C or 33°C. The cells were then treated with IFNα and infected with CHIKV. At 37°C, 50% protection against CHIKV-induced cytopathic effects (CPE) required ≈1.5 U/ml of IFNα (Fig 2H). At 35°C, ≈50 U/ml of IFNα were required, and at 33°C no significant protection against CPE was observed even at 100 U/ml (Fig 2H). These data are consistent with Fig 2G and other *in vitro* data using different mammalian viruses [4–6] and argue that type I IFN receptor signaling and/or downstream anti-viral responses are diminished at the lower temperatures. In the absence of IFNα, CHIKV replication in Vero cells was not significantly affected by these small temperature changes (Fig 2I).

### Ambient temperature mediates its effects early in infection

**Fig 3:**
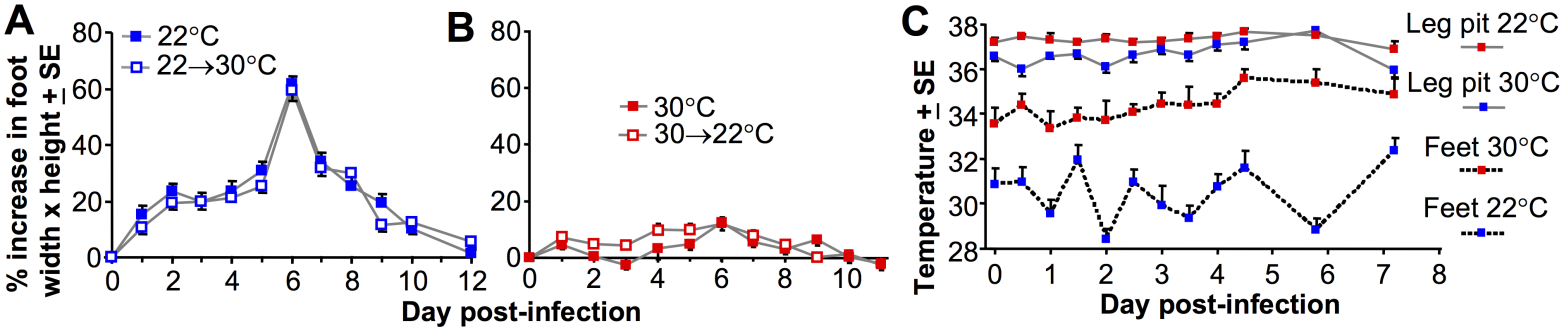
Moving mice from 22°C to 30°C and vice versa. (A) Foot swelling in mice infected s.c. in the feet and held at 22°C (22°C) or held at 22°C and then at the end of day 4 post infection moved to 30°C (22°C→30°C); (n = 12 feet from 6 mice per group). (B) Foot swelling in mice infected s.c. in the feet and held at 30°C (30°C) or held at 30°C and then at the end of day 4 post infection moved to 22°C (30°C→22°C); (n = 12 feet from 6 mice per group). (C) Temperature measurements were taken in the rear leg pits and the surface of feet of mice housed at 22°C or 30°C on the indicated days post s.c. infection in the feet (n = 6 mice per group). Measurements were taken using a pediatric infra-red thermometer, and were taken in quadruplicate to produce a mean for each mouse, with the mean of 6 mice and SE shown.

Type I IFN protein levels rise rapidly and fall in CHIKV-infected C57BL/6 mice within 4 days [32,41], with this transient cytokine production key to generating protective innate anti-viral responses [27,41]. When CHIKV-infected C57BL/6 mice were housed at 22°C and then moved to 30°C at the end of day 4 post-infection (the end of the viremic period), foot swelling was the same as that seen in mice housed at 22°C throughout (Fig 3A). Similarly when C57BL/6 mice were housed at 30°C and then moved to 22°C on day 4 post-infection, the reduction in foot swelling was similar to that of mice housed at 30°C throughout (Fig 3B). The reduced arthritic disease seen at 30°C was therefore due to effects mediated within the first 4 days post infection, the period of significant IFNα/β production and peak viral replication. These experiments (i) support the view that the ambient temperature mediates its effects during the time of IFNα/β production, and (ii) argue that any warming treatment attempting to reduce arthritic disease [1,2] likely needs to be initiated very early in infection.

### The temperature phenotype was not associated with a detectable fever

The potential benefits of fever for fighting infections have been suggested in various contexts [9,10]. C57BL/6 mice housed at 22°C and infected with CHIKV do not develop a detectable fever [41], whereas most humans develop a fever after CHIKV infection [50]. To determine whether housing mice at 30°C promotes detectable fever development, estimates of core temperatures were obtained using a pediatric infra-red thermometer gently pressed into the pit of the back leg of a restrained mouse for 10 seconds, with the leg folded over the end of the probe. No significant fever was detected in mice kept at 30°C or 22°C (Fig 3C). In humans, fever coincides with peak viremia [50], which would be day 1–2 post infection in mice (Fig 1A). As might be expected for a homeotherm, the core temperature measurements were only marginally higher at 30°C (Fig 3C).

Temperature readings were also taken from feet by placing the thermometer onto the hairless skin regions of the hind foot, with mice housed at 30°C showing ≈3–4°C higher foot skin temperatures than mice housed at 22°C (Fig 3C, repeat measures ANOVA p<0.001). After day 4, foot skin temperatures for mice housed at 30°C did show a ≈1°C increase relative to day 0 (from ≈34°C to ≈35°C), presumably as a consequence of the CHIKV-induced arthritic inflammation (calor).

Thus for mice housed at 22°C, feet were about ≈6°C cooler than core, whereas for mice housed at 30°C feet were ≈2°C cooler than core. Similar differences in skin temperatures at the trunk and the extremities have been reported in healthy humans placed in rooms kept at 30°C or 22°C [51].

### Intraperitoneal inoculations of CHIKV

**Fig 4:**
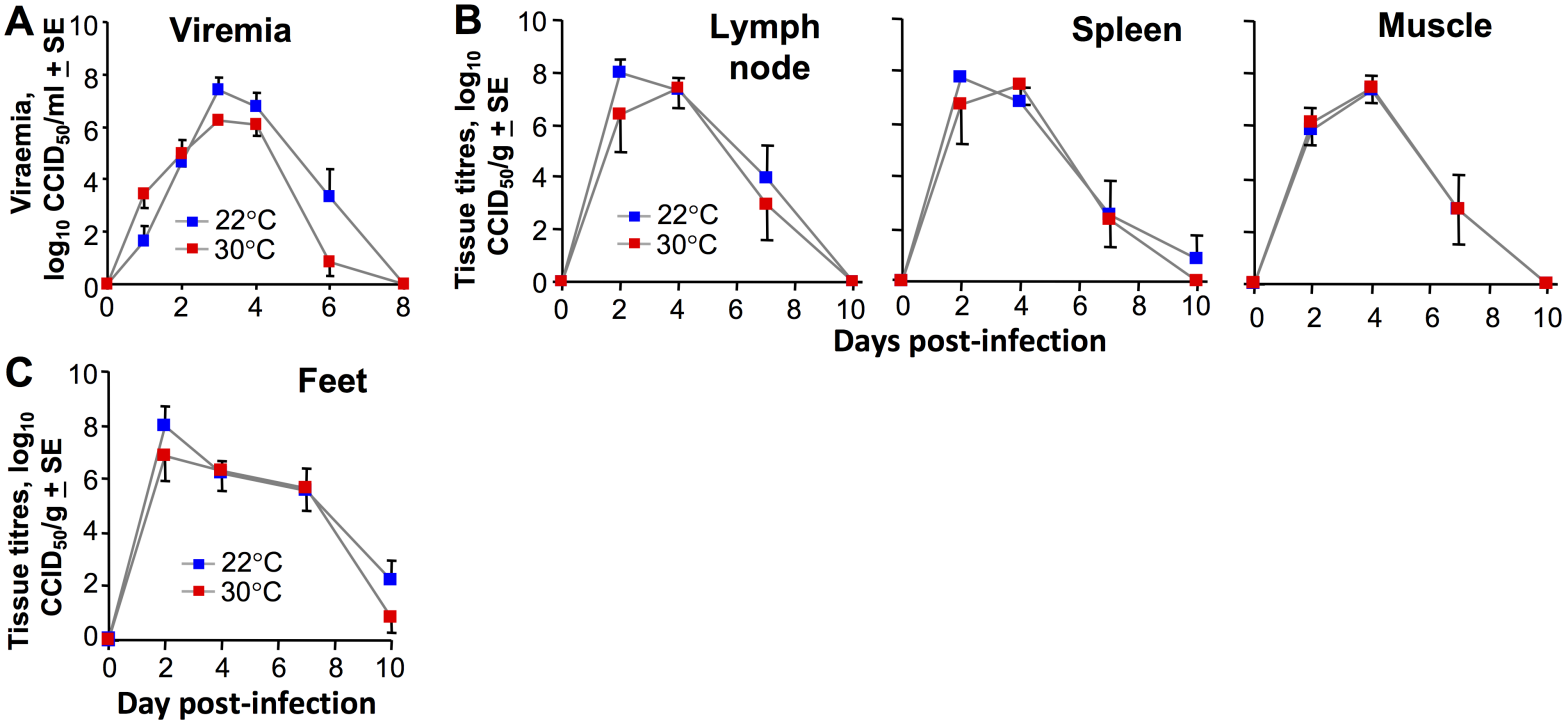
Intraperitoneal inoculation of CHIKV. Mice were housed at 22°C or 30°C and were infected i.p. with CHIKV. (A) Viremia (n = 6 per group). (B) Tissue titers (n = 6 per group, as in Fig 1). (C) Foot tissue titers (n = 6 mice, 12 feet per group).

The mice in Fig 1 were inoculated with CHIKV s.c. into the feet, with feet ≈3–4°C cooler in mice housed at 22°C when compared with mice housed at 30°C (Fig 3C). As feet represent a major site of virus replication after s.c. CHIKV inoculation into the feet (Fig 1A) [32], mice were injected with CHIKV via the intra-peritoneal (i.p.) route, to ascertain whether the temperature phenotype was dependent on the route of inoculation. Although the viremia showed a hint of reduced viral load at 30°C, no significant differences in viremia (Fig 4A), tissue titers (Fig 4B) or feet titers (Fig 4C) for mice housed at the two temperatures was seen after i.p. inoculation. This contrasts with s.c. inoculation; viremia (Fig 1H), tissue titers (Fig 1I) and feet titers (Fig 1A). (Foot swelling does not occur after i.p. inoculation of CHIKV [32]).

Thus the temperature phenotype (described in Fig 1) seen after inoculation of CHIKV into cooler feet (Fig 3C) is lost when virus is inoculated via the i.p. route. After i.p. inoculation, initially virus replication likely occurs at core temperatures, which were similar for mice housed at 30°C or 22°C (Fig 3C). In the absence of a temperature difference, type I IFN production would not be different and systemic anti-viral activity remains largely unaffected (Fig 4A–4C). Although viral inoculation via the i.p. route is unlikely to occur naturally, the experiment illustrates that the increased CHIKV replication seen in mice housed at 22°C (Fig 1) requires that initial viral replication occurs in tissues that are slightly cooler than the core temperature.

### The RRV model

**Fig 5:**
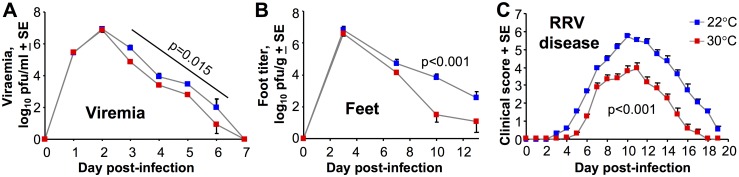
Affect of ambient temperature in the RRV model of alphaviral arthropathy. Mice were housed at 22°C or 30°C and were infected s.c. in the pectoral region with RRV. (A) Viremia. (Statistics by repeat measures ANOVA for days 3–6, n = 5 mice per group). (B) Feet titers. (Statistics by Mann-Whitney U test, n = 5 feet from 5 mice per group). (C) Disease severity score. This is primarily a score of hind limb paralysis. (Statistics by repeat measures ANOVA, n = 5–10 mice per group, day 0–21).

Arbovirus disease models have generally been optimized with respect to route and dose, and the RRV mouse model of arthropathy involves inoculation of virus s.c. into the pectoral area of young mice, with infection showing tropism for tissues in the hind limb [33]. This model might thus be viewed as being an intermediate between the foot s.c. and i.p. routes of inoculation described above. After viral inoculation the viremia in RRV infected mice was ≈0.5–1 log lower on days 3–6 for mice housed at 30°C, although this was significant only when a repeat measures ANOVA was applied (Fig 5A). Foot titers started to show slight differences days 3 and 7, but differences only reached significance on day 10 post RRV infection when titers were ≈2 logs lower in mice housed at 30°C (Fig 5B). The disease severity was significantly lower for mice held at 30°C, with differences in disease scores increasing after day 8 (Fig 5C). The disease severity scoring in this model is primarily derived from assessing hind limb weakness [34]. Thus in a model where viral tropism results in hind limbs becoming a major site of viral replication and disease, a clearly significant reduction in foot titers and disease was again evident for mice housed at 30°C. One might of course now argue that viral tropism in this model is due (at least in part) to the lower limb temperatures and the resulting reduction in type I IFN activity [52].

### RNA-Seq and bioinformatics after poly(I:C)/jetPEI inoculation into feet

The results so far suggest that for wild-type mice housed at 30°C and infected in the feet, alphaviral replication in limbs and extremities is more effectively controlled by type I IFN responses. As a consequence, viral replication and arthritic disease is significantly reduced. To investigate the role of ambient temperature on type I IFN responses in the periphery, RNA-Seq was undertaken on mRNA from four samples (i) feet from naive control mice held at 30°C (30C), (ii) feet from mice held at 30°C treated with poly(I:C)/jetPEI for 12 h (30T), (iii) feet from naive control mice held at 22°C (22C), and (iv) feet from mice held at 22°C treated with poly(I:C)/jetPEI for 12 h (22T). As described above, injecting poly(I:C)/jetPEI into feet represents a model of dsRNA-induced arthritis [47], and ensures an equivalent amount of dsRNA is present in the feet of mice housed at the two temperatures.

Quality control analyses for the RNA-Seq are shown in S3 Fig. DEGs (FDR q<0.01 and CPM>1 in at least 3 samples) are shown in S1 Table. Global analyses of DEGs illustrated, for instance, that genes regulated by poly(I:C)/jetPEI were qualitatively, if not quantitatively, similar at the two temperatures, with 22C vs 22T and 30C vs 30T showing considerable overlap (S4 Fig). As might be expected (i) most of the up-regulated genes were ISGs (S4 Fig) and (ii) the Molecular and Cellular Functions feature of IPA clearly illustrated that genes associated with cell growth, survival, movement, morphology and development were up-regulated in feet of mice housed at 30°C in the presence or absence of poly(I:C)/jetPEI treatment (S2 Table).

Gene set enrichment analyses illustrated a highly significant concordance between up and down regulated DEGs after poly(I:C)/jetPEI treatment verses up and down regulated DEGs after CHIKV infection (S5A Fig). In addition, at both temperatures, >79% of ISGs up-regulated by poly(I:C)/jetPEI were also up-regulated by CHIKV (S5B Fig). These analyses illustrate that poly(I:C)/jetPEI injection and CHIKV infection have very similar gene expression signatures.

**Fig 6:**
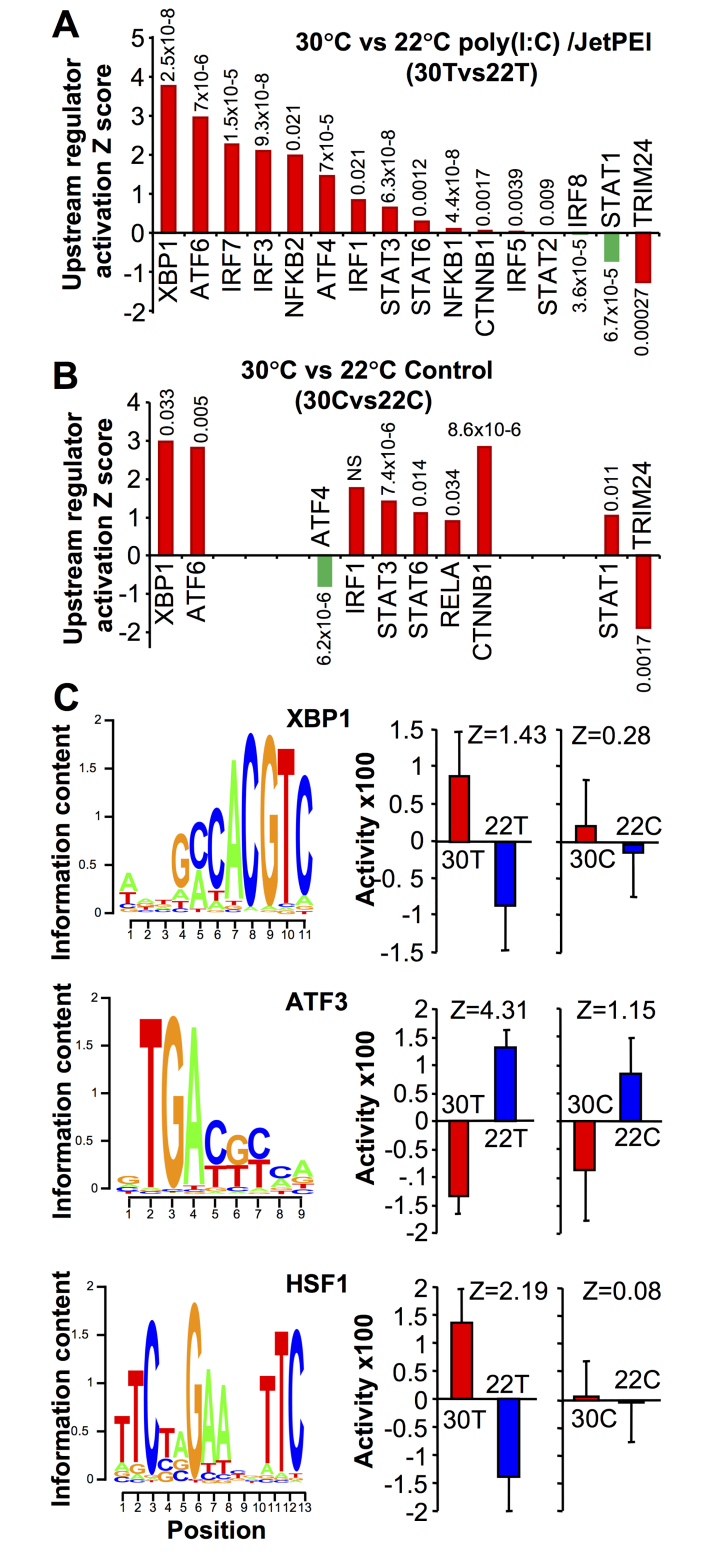
RNA-Seq of feet of mice housed at 30°C or 22°C. (A) Mice housed at 30°C or 22°C were injected s.c. into feet with poly(I:C)/jetPEI. After 12 hours feet were harvested, mRNA was extracted and subjected to RNA-Seq analysis. Differentially expressed genes (q<0.01, CPM>1 in at least 3 samples) were analyzed by the IPA upstream regulator (USR) function (direct only). Selected USRs associated with the type I IFN response are shown ranked by Z score. Transcription factor activities associated with enhanced type I IFN responses are colored red. Transcription factor activities associated with reduced type I IFN responses are colored green. P values are provided above/below the bars. (B) As for (a) with mice housed at 30°C or 22°C for 2 days, but with feet harvested without any treatment. (C) The raw RNA-Seq sequencing data was independently analyzed by ISMARA (full results shown in S1 Table). The transcription factor binding site (left) and site activity (right) are shown (with associated Z values) for XBP1, ATF3 and HSF1. ISMARA quantifies significance as a Z-value, with a site with a higher Z value ranked as more significant than one with a lower Z value. The activity score is an arbitrary score provided by the program and if error bars overlap significance is deemed not to have been reached [54]. The significant Expression/Activity correlations (positive for XBP1 and HSF1, and negative for ATF3) are shown in S7B Fig.

Comparing 30T vs 22T (using both up and down regulated genes) and the upstream regulator (USR) function of IPA (direct only), a series of USRs were identified. Prominent among those more active at 30°C were USRs associated with the unfolded protein response (UPR), specifically XBP1, ATF6, and ATF4 (Fig 6A). mRNA levels of XBP1 (the most prominent USR) were also significantly higher at 30°C, although the increase in spliced XBP1 was not significant (S6A Fig).

Importantly, two transcription factors central to the type I IFN response to CHIKV, IRF3 and IRF7 [41], were more active at 30°C, with USRs showing high activation Z scores (>2) and significance for 30T vs 22T (Fig 6A). Other USRs associated with the type I IFN response were also identified; NF-кB (NFKB1/2), IRF1, STAT3, STAT6, IRF5 and STAT2 (Fig 6A), with most of these also activated during CHIKV infection [27]. β-catenin (CTNNB1) was also recently shown to regulate RIG-I-dependent responses [53]. Only IRF8 and STAT1 [27] showed lower activity at 30°C. TRIM24, a negative regulator of the type I IFN response showed lower activity at 30°C (Fig 6A). The USR analysis showed no indication of a fever signature, consistent with data in Fig 3C. Multiple USRs associated with promotion of the UPR and type I IFN responses were thus more active in feet of mice housed at 30°C.

After poly(I:C)/jetPEI treatment the mRNA expression of a number of key genes associated with the type I IFN response were also more up-regulated at 30°C than at 22°C, including IRF7, Oas2 and Oas3 (S6B Fig). However, mRNA levels for IFNβ were not significantly different for 30T vs 22T (S6C Fig), consistent with the data in Fig 2G. (There were insufficient reads for the IFNαs to make any comparisons; unique reads mapping to IFNα subtypes are inherently low even after CHIKV infection [27]).

An identical RNA-Seq and IPA USR analysis to that described above was conducted using feet from naïve untreated mice held at 30°C or 22°C for 5 days (30C vs 22C) (Fig 6B). XBP1 and ATF6 (UPR-associated USRs) were again identified with high Activation Z scores. A number of other USRs associated with promotion of the type I IFN response were also more active at 30°C, whereas IRF3, IRF7 (and IRF5) were not identified (Fig 6B).

### Promoter activity analysis using ISMARA

A recently released program called “Integrated System for Motif Activity Response Analysis” (ISMARA) [54] allows for an independent analysis of the RNA-Seq data and provides an activity score for known promoters. The majority of the transcription factors identified as more active at 30°C by the IPA USR (Fig 6A and 6B) also showed a trend towards increased activity in the ISMARA (S7A Fig). Importantly, prominent in both the IPA USR (Fig 6A and 6B) and the ISMARA outputs (Fig 6C, S1 Table), was XBP1 (a key player in the UPR), with the ISMARA showing a clear increase in XBP1 promoter activity at 30°C after poly(I:C)/jetPEI treatment (Fig 6C, XBP1 30T vs 22T). The same trend (although not significant) was also seen for 30C vs 22C (Fig 6C, XBP1 30C vs 22C). The spliced form of XBP1 is transcriptionally active [55].

The top scoring site (by Z score) in the ISMARA output for 30T vs 22T was ATF3, with ATF3 showing reduced activity in feet of mice housed at 30°C (Fig 6C, S1 Table). ATF3 was also prominent (although less significant) for 30C vs 22C (Fig 6C, S1 Table). ATF3 is well described as a stress-responsive transcription factor [56], and has been associated with repression of type I ISGs [57] (and proinflammatory cytokine expression [58,59]). The reduced ATF3 activity at 30°C (Fig 6C) is thus consistent with increased antiviral activity in feet of mice housed at 30°C. As expected for a repressor, the ISMARA showed a negative Expression/Activity correlation for ATF3 (rho = -0.76, p = 0.007), whereas XBP1 and HSF1 showed significant positive correlations (S7B Fig). Further supportive evidence for a role for ATF3 is provided by the observation that a number of ISGs up-regulated in ATF3^-/-^ fibroblasts *in vitro* [57] were also up-regulated in feet at 30°C after poly(I:C)/jetPEI treatment (RNA-Seq, p<0.05); specifically IRF7, BST2, SLFN1, FPR2, LY6A, OAS1G, TRIM30D, FCGR4, SIGLEC1, PLAC8, TLR1, SELL, IFITM6, FPR2 AND NUPR1.

The ISMARA also highlighted increased heat shock factor 1 (HSF1) activity after poly(I:C)/jetPEI treatment for mice held at 30°C (Fig 6C), with a number of heat shock proteins identified as targets of HSF1 (S7C Fig). HSF1 activity is induced during conditions that cause an increase in unfolded or misfolded proteins, primarily in the cytoplasm and nucleus [60]. Such conditions include, but are not limited to heat shock [61], with HSF1 believed to control transcription of genes dedicated to restoring protein-folding homeostasis [62].

## Discussion

At room temperature (22°C) limbs and extremities in humans are usually cooler than core temperatures [51], and alphaviral arthropathies primarily affect joints in limbs and the extremities [23,25,26]; the marked concordance is illustrated in S8 Fig. The data presented herein argues that a major reason why alphaviral arthropathy primarily affects these joints is that these tissues are often cooler, resulting in reduced type I IFN anti-viral responses, increased alphaviral replication and thus exacerbated arthropathy. A number of other viral arthropathies have similarly been shown to predominantly affect the extremities, including dengue [63], rubella [64], parvovirus [65], and hepatitis B [66], suggesting this affect is not restricted to alphaviruses.

The results suggest that limb temperatures can have profound effects on alphaviral infection and disease. The implications might be that the Elizabeth Kenny limb warming therapy [1,2] might find utility for treating alphaviral arthritides. However, such treatment might need to start very early post onset of viremia, before the virus has had a chance to seed and substantially replicate in joint tissues. Applying “heat therapy” 4 days post infection in mice was already too late to change the course of arthritic disease (Fig 3A). In humans, fever (often accompanied with joint pain) is usually the first sign of CHIKV infection, by which time viremia has usually already peaked [50] and joints have likely already become infected. Whether warming of peripheral joints at this stage would lead to significant alterations in the course of this often chronic condition [30,67] remains unclear. Warming treatment initiated after the acute phase may have little impact, as the virus may no longer be replicating and sensitive to anti-viral type I IFN responses [36,68].

The data might argue that alphaviral arthropathies should be overtly less severe in people living in tropical climates; however, establishing such an association would likely be confounded by a number of factors including (i) air conditioning [69], (ii) lack of application of validated instruments for defining disease severity and lack of accounting for co-morbidities [70], (iii) different levels of co-morbidities (often associated with severe disease) [71], (iv) different age structures with the elderly prone to more severe disease, (v) suboptimal diagnosis (e.g. differentiation from *inter alia* dengue and rheumatoid arthritis) [72], (vi) different access to non-steroidal anti-inflammatory drugs and/or paracetamol (acetaminophen), (vii) viral genotypes with different pathogenicities and geographical distributions [73] and (viii) different mosquito vectors and vector competencies [74,75].

We show herein that the augmented type I IFN response for mice housed at 30°C correlated with an increase in UPR-associated transcriptional signatures. That the UPR can augment (or improve priming of) the type I IFN response has been reported by several groups in a variety of settings [53,76–82]. Network analyses also support the view that the UPR and anti-viral pathways intersect at several nodes (S9 Fig). XBP1 (dominant in Fig 6A, 6B and 6C) has also been associated with improved type I IFN response in several settings [83–88]. Some proteins are also reported to be thermolabile even at 37°C [89,90], particularly when other stressors are present [91], potentially contributing to UPR activation. Unfortunately, measuring the thermostability of cellular proteins in eukaryotic cells *in vivo* remains challenging [92], although reducing temperature to increase protein stability is a well understood concept [93].

Exactly how the UPR and/or XBP1 might promote type I IFN responses remains unclear, with XBP1 binding to the IFNβ promoter [87] perhaps unlikely in the current setting (S6C Fig, Fig 2G). A role for GADD34 [94,95] is also not supported by the above and GADD34 expression data (S10 Fig). We (like others [84]) have also been unable to find a significant correlation between XBP1 splicing [96] and elevated type I IFN responses (S6A Fig), although this might require examination of certain cell types [83,86] and/or sampling over a specific time period. The UPR may promote and/or prime type I IFN responses via the IRE1-RIDD-RIG-I pathway [80,81], a pathway for which XBP1 splicing is dispensable [88]; (RIDD stands for IRE1-dependent decay of mRNA). The UPR sensor IRE1α (as well as splicing XBP1) degrades RNA, with the products detected by RIG-I [88], a RNA sensor that also binds CHIKV RNA [97]. IRE1α may also be ancestrally related to the anti-viral effector RNase L [85,98]. However, until a mechanism can be elucidated in the current setting, the UPR and promotion of the type I IFN response remains a correlation, with other mechanisms potentially involved [99,100].

The findings presented herein raise a number of questions; for instance, does the reduced body temperature in the elderly [101], especially those >75 years of age [102], contribute to the increased CHIKV-associated mortality seen in such patients [103,104]? Is the likelihood of severe CHKV disease increased if the infectious mosquito bite occurs at cooler extremities [105]? Can CHIKV-induced fever in humans increase the temperature of limbs and extremities in time to increase the local type I IFN responses and ameliorate arthropathy? That different ambient temperatures can affect anti-viral type I IFN responses *in vivo*, even in homeotherms, extends *in vitro* data [4–6] and evidence from poikilotherms [14,16], and may have implications for understanding other human diseases believed to be influenced by environmental temperatures [106–108].

## Methods

### Ethics statement

All mouse work was conducted in accordance with the “Australian code for the care and use of animals for scientific purposes” as defined by the National Health and Medical Research Council of Australia. Mouse work was approved by the QIMR Berghofer Medical Research Institute animal ethics committee (P1060 A705603M) and was conducted in a biosafety level-3 facility at the QIMR Berghofer MRI. Mice were euthanized using carbon dioxide.

### Mice and CHIKV infection

Female C57BL/6J mice (6–8 weeks) were purchased from Animal Resources Center (Canning Vale, WA, Australia). IRF3/7^-/-^ mice were kindly provided by M. S. Diamond (Washington University School of Medicine, St. Louis, MO) and were bred in house at QIMR B [41]. IFNAR1^-/-^ mice on a C57BL/6 background [109] were supplied by Dr P Hertzog (Monash University, VIC, Australia) and bred in house. Female mice were inoculated with 10^4^ CCID_50_ of the Reunion Island isolate (LR2006-OPY1; GenBank KT449801 [36]) (i) i.p. or (ii) s.c. into each hind foot as described previously [32,36]. Serum viremia, tissue titers and foot swelling were determined as described [32,36].

### Histology and immunohistochemistry

Histology (H&E) and immunohistochemistry was undertaken on formalin fixed, decalcified feet using an anti-CHIKV capsid monoclonal antibody as described [44]. Slides were scanned using Aperio AT Turbo (Aperio, Vista, CA) and analyzed using Aperio ImageScope software (v10) and the Positive Pixel Count v9 algorithm using default settings.

### Ross River virus model

Female 21 day old C57BL/6J mice were inoculated s.c. in the pectoral area with 10^3^ plaque-forming units (PFU) of RRV (T48 strain) as described [34]. Disease was determined by assessing grip strength and gait, and scored as follows: 0 = no disease; 1 = ruffled fur, 2 = very mild hind limb weakness; 3 = mild hind limb weakness; 4 = moderate hind limb weakness; 5 = severe hind limb weakness/dragging; 6 = complete loss of hind limb function, as described [34].

### Temperature measurements

Temperature measurements were taken using a pediatric infra-red ear thermometer (Welch Allyn Pro 4000, Braun Kronberg Type 6021 Hechingen, Germany). The thermometer probe was gently pressed into the pit of the back leg of a restrained mouse for 10 seconds (gently folding and holding the leg over the end of the probe) before a temperature measurement was taken. This was repeated on the other leg, and the procedure repeated to obtain a mean of four measurements. Temperature readings were also taken from the feet by placing the thermometer probe onto the largely hairless (walking-pad free) regions of the hind foot; this was repeated on the other leg, repeated, and a mean of four measurements obtained. Four measurements (two on the right, two on the left) were thus taken to produce a mean for each mouse for both leg pits and feet. The mean SD across the data set for quadruplicates was 1.04°C.

### Serum IFNα/β measurements

Serum was collected in Microvette 500 Z-gel tubes (Sarstedt, Germany) and cytokines measured using IFN alpha/IFN beta 2-Plex Mouse ProcartaPlex Panel (Thermofisher), with beads run on a BD LSR Fortessa 4 and data analyzed using BD FACSDiva (v8.0.1) and FCAP Array software (V3.01).

### *In vitro* Vero experiments

Vero cells (ATCC CCL-81) were seeded into 96 well plates and cultured for 24 hrs at the indicated temperatures and treated with human IFNα (Sigma I 2396; a mixture of 10 IFNα species) for 4 hrs. CHIKV was added (MOI = 0.05) and incubation continued at the same temperature. Cytopathic effect (CPE) was assessed by crystal violet staining after 3 days. For virus replication Vero cells after seeding at the indicated temperatures in triplicate in 24 well plates were infected with CHIKV for 6 hrs, were washed and then incubated at the same temperatures. Viral titers in the supernatants were assessed at the indicated times after washing.

### RNA-Seq

Female C57BL/6J mice (6–8 weeks old) were housed at 30±1°C or 22±1°C for 5 days. Mice were left untreated (Control) or feet were injected s.c. (as for CHIKV infection [32]) with 0.5 μg of poly(I:C) mixed with jetPEI transfection reagent (Polyplus Transfection, NY, USA) [47] (Treated). Twelve samples were prepared; each contained pooled RNA from 3–4 mouse feet from 3–4 different mice, with equal amounts of RNA from each foot in each pool. The 12 samples represent 3 biological replicates for each of the four conditions; (i) feet from control (C) mice housed at 30°C (30C), (ii) feet from control (C) mice housed at 22°C (22C), (iii) feet from poly(I:C)/jetPEI treated (T) mice held at 30°C (30T), (iv) feet from poly(I:C) /jetPEI treated (T) mice held at 22°C (22T). Sample preparation and RNA-Seq was undertaken essentially as described [27]. Briefly, feet were lacerated and placed in RNAlater (Life Technologies), homogenized in TRIzol (Invitrogen), centrifuged (12,000 g x 10 min) and RNA extracted from the supernatants as per manufacturer’s instructions. RNA samples were DNase treated using RNAse-Free DNAse Set (Qiagen) and purified using an RNeasy MinElute Kit (Qiagen). Samples were sent to the Australian Genome Research Facility (Melbourne, Australia). cDNA libraries were prepared using a TruSeq RNA Sample Prep Kit (v2) (Illumina Inc. San Diego, USA), which includes isolation of poly-adenylated RNA using oligo-dt beads. cDNA libraries were sequenced (100 bp single end reads) using the Illumina HiSeq 2500 Sequencer (Illumina Inc.). The per base sequence quality was high, with >84% of bases above Q30 for all 12 samples. The primary sequence data was generated using the Illumina bcl2fastq 2.18.0.12 pipeline. After removal of Illumina adaptor/over-represented sequences and cross-species contamination, reads were mapped to the mouse genome (Mus_musculus.GRCm38) using Tophat aligner (v2.0.14). The counts of reads mapping to each known gene (with gencode annotation vM6 as reference) were summarized at gene level using the featreCounts v1.4.6-p5 utility of the subread package (http://subread.sourceforge.net/). The transcripts were assembled with the Stringtie tool v1.2.4 (http://ccb.jhu.edu/software/stringtie/) utilizing the read alignment and reference annotation based assembly option (RABT).

The read counts were used to determine gene expression and identify differentially expressed genes (DEGs) using R packages (R version 3.2.0) ‘edgeR’ (v3.10.5) and ‘limma’ (3.24.15). (https://bioconductor.org/packages/release/bioc/html/edgeR.html). The default TMM normalization method of edgeR was used to normalize the counts. The GLM model was used to perform differential expression comparison between the groups. Genes that had >1 CPM in at least 3 samples are retained for the further analysis. Differentially gene expression was considered significant if the Benjamini-Hochberg corrected p-value (i.e. FDR or q value) was <0.01. DEGs were analyzed by Ingenuity as described [27] and ISMARA [54] by uploading the RNA-Seq fastq files, identifying the replicates (allowing averaging, n = 3) and undertaking pair-wise comparisons (30C vs 22C and 30T vs 22T).

### Statistics

Statistical treatment of mouse data was performed using IBM SPSS Statistics (version19). The t test was used if the difference in the variances was <4, skewness was >-2, and kurtosis was <2; where the data was nonparametric and difference in variances was <4, the Mann Whitney U test was used, if >4 the Kolmogorov-Smirnov test was used [36]. The repeat measures ANOVA was used for RRV data.

## Supporting information

S1 Fig qRT PCR and immunohistochemistry. (A) qRT PCR for CHIKV E1 of feet of mice held at 22°C and 30°C. C57BL/6 mice were infected s.c. in the feet as for Fig 1 and at the indicated times feet were harvested (n = 3 feet from 3 mice per time point and temperature) and CHIKV E1 RNA levels determined by qRT PCR as in Fig 1B. Statistics by t test. (B) Immunohistochemistry as in Fig 1F on mock infected mouse tissues.
(PDF)

S2 Fig Serum IFNα levels and responses after CHIKV infection. (A) Mice were housed at 22°C or 30°C (n = 5/6 per group) and were infected s.c. in the feet with CHIKV as in Fig 1. Serum IFNα levels were then determined at the indicated times post infection. The numbers above the bars represent the mean viremias in log_10_CCID_50_/ml. On days 2 and 3 the mean viremias were 3–4 logs higher in mice housed at 22°C. (B) RNA-Seq analysis of day 2 feet from mice infected with CHIKV was performed as described [27], with mice housed at 30°C or 22°C. DEGs for both temperatures were determined relative to mock infected mice housed at the same temperature. DEGs (q<0.01, fold change >2) were analyzed using the Upstream Regulator (USR) feature of Ingenuity Pathway analysis (IPA). As expected, given the higher viremia in mice housed at 22°C (see above), most USR pathways associated with the type I IFN response had higher Z scores for mice housed at 22°C than for mice housed at 30°C. However, despite the difference in viral loads, USR Z scores for well annotated IFNαs that are involved in the secondary amplification loop were actually higher for mice housed at 30°C than for mice housed at 22°C. (C) Using the same DEG lists as in B and the “Top Diseases and Bio Functions” feature of IPA, “Immunological Disease” and “Cellular Movement” showed higher significance (lower p value ranges) for mice housed at 22°C than for mice housed at 30°C. This is consistent with the H&E data shown in Fig 1.
(PDF)

S3 Fig RNA-Seq quality control analyses. (A) Library sizes. (T refers to Treatment with poly(I:C)/jetPEI and C to no treatment Controls). (B) Read numbers, data yield and percentage of reads mapping to the mouse genome. The per base sequence quality was high, with >84% of bases above Q30 for all 12 samples. (C) MDS clustering for all samples, and for 30T vs 22T samples. (D) Boxplots showing the distribution of expression values for all samples before and after normalization. (E) Smear plots: log-fold-change plotted against the log counts per million. Genes showing significant differences (FDR<0.05) are in red. Blue lines represent log2 fold change of 1 and -1 (i.e. fold change of 2 and -2).
(PDF)

S4 Fig Global analysis of DEGs. Overlaps of DEGs (FDR<0.01, CPM>1 in at least 3 samples) for 30Cvs22C, 30Tvs22T, 22Cvs22T and 30Cvs30T are show for up and down regulated genes. Type I interferon stimulated genes (ISGs) were determined by Interferome (v2.0), selecting type I interferon and default settings (All) for all other search conditions.
(PDF)

S5 Fig Similarities in gene expression profiles after poly(I:C)/jetPEI injection and CHIKV infection. (A) Gene set enrichment analyses (GSEA 3.0, Broad Institute) comparing DEGs (fold change >2, q <0.01) 12 hours after poly(I:C)/jetPEI injection (as in S4 Fig) and 2 days after CHIKV infection (as in S2B Fig). (B) ISGs significantly induced (fold change >2, q <0.01) on day 2 post CHIKV infection [27] show considerable overlap with ISGs significantly induced 12 hours after poly(I:C)/jetPEI injection. ISGs were identified via Interferome (selecting type I interferon and default settings ‘All’ for all other search conditions).
(PDF)

S6 Fig Expression of selected genes associated with anti-viral activities. (A) XBP1 mRNA levels after poly(I:C)/jetPEI injection of mice housed at 22°C or 30°C derived from the RNA-Seq data (Statistics by t tests). Spliced XBP1 levels were determined using the Sashimi feature of the Integrative Genome Viewer (IGV) v2.3.34. (B) Using the DEG lists for 30T vs 22T (top graph) and 30C vs 22C (bottom graph) (q<0.01) the mean fold change (no log transformation applied) for selected genes where the change in expression would be associated with promotion of anti-viral activities (red) or where the change in expression would be associated with reduction of anti-viral activities (green). IRF7 is bolded. (C) IFNβ mRNA expression. Normalized counts for IFNβ mRNA for each condition; n = 3 for each. Poly(I:C)/JetPEI induced IFNβ mRNA expression at both temperatures (i.e. 22C vs 22T and 30C vs 30T), but levels were not significantly different for 30T and 22T.
(PDF)

S7 Fig ISMARA. (A) Activity and Z scores for promoter site activities for selected transcription factors associated with the type I interferon response. Analyses as for Fig 6C. The activity of the indicated transcription factors nearly always tended to be higher at 30°C, although only XBP1 and ATF3 (see Fig 6C) reached Z scores >2 and non-overlapping error bars, conditions for clear significance recommend by ISMARA protocols. A unique IRF7 site is not available in ISMARA. (B) Expression/Activity correlations. ISMARA Expression/Activity correlations showing Pearson’s correlation coefficients (r) and p values for transcription factor sites shown in Fig 6C. (C) HSF1 target genes (identified by ISMARA) for HSF1 for 30T vs 22T, with heat shock proteins highlighted in yellow.
(PDF)

S8 Fig Joints at lower temperatures are commonly affected during alphaviral arthropathies. (A) Limb and body temperatures in humans (adapted from New Human Physiology 2^nd^ Edition. Chapter 21: Thermo-Regulation, Temperature and Radiation. Eds. Paulev PE, Zubieta-Calleja G. Copenhagen, Denmark). (B) The number of patients (n) and the percentage of CHIKV patients (%) with arthropathy in the indicated joints (n = 71 patients) (taken from Borgherini *et al*., 2007 [26]). (C) The percentage of RRV patients with arthritic disease in the indicated joints (for example; 69% of RRV patients report pain and/or swelling in their wrist joints) (produced by Environmental Health Directorate, Dept. Health, WA, Australia, 2006). (D) Joints affected in Mayaro virus patients (taken from Halsey *et al*., 2013 [25]). M03, M06 and M12 represent 3, 6, and 12 months follow-up. The numerator represents the number of patients reporting arthropathy in the indicated joints, the denominator the number of patients present at the indicated follow-up time. The percentage is numerator/denominator x100.
(PDF)

S9 Fig Network analyses. Using the commercial GeneGo MetaCore software (http://portal.genego.com), genes associated with (i) the unfolded protein response (UPR) (blue) and (ii) Type I IFN response together with Response to RNA virus (green) were uploaded (GO module). Networks were constructed and merged (Build network module) and expression data from (A) 30Cvs22C or (B) 30Tvs22T applied as a filter to the merged networks. The two networks intersect at a number of nodes, and even without treatment feet of mice housed at 30°C show up-regulation (red circles) of genes associated with anti-viral responses.
(PDF)

S10 Fig GADD34 (Ppp1r15a) mRNA expression after CHIKV infection and dsRNA treatment. A major consequence of the UPR and responses to dsRNA (via PKR [110]) is translation inhibition, which appears to promote anti-viral responses via reduced translation of inhibitors (e.g. A20, SHIP-1, IкBα) [94]. GADD34-mediated relief of translational repression (via eIF2α dephosphorylation and/or stress granule dissolution) is required for IFNβ protein synthesis [94,95]. (A) GADD34 mRNA is up-regulated in feet and lymph nodes 2 days post CHIKV infection (using the RNA-Seq data set described in Wilson *et al* 2017); a result consistent with [95]. (B) Although GADD34 mRNA was also up-regulated in feet at 12 h after poly(I:C)/JetPEI injection, there were no significant differences for mice housed at 30°C.
(PDF)

S1 Table DEG lists. Differentially expressed genes identified by RNA-Seq comparing 22C and 30C, and 22T and 30T (C for Control, T for poly(I:C)/jetPEI Treatment). For all DEGs FDR<0.01, and CPM>1 in at least 3 samples (3 replicates for each of the four conditions). The complete IPA upstream regulator (USR) and ISMARA analyses described in Fig 6 are also provided (key regulators are shaded in yellow).
(XLSX)

S2 Table Molecular and Cellular Functions analyses. Genes associated with cell growth, survival, movement, morphology and development are up-regulated in feet of mice held at 30°C both in the absence of treatment (30C vs 22C) and after poly(I:C)/jetPEI treatment (30T vs 22T). IPA “Molecular and Cellular Functions” analysis (direct only) for all DEGs (up and down regulated) was undertaken for 30Cvs22C and 30Tvs22T (FDR<0.01, CPM >1 in at least 3 samples). (“Molecular and Cellular Functions” is a sub-category of the “Diseases and Functions” feature, which returns a number of “Diseases or Functions Annotations” for each of the listed categories; thus providing a p value range).
(DOCX)
