# Supplementary figures and images for: Lower temperatures reduce type I interferon activity and promote alphaviral arthritis
Source: PLoS Pathog. 2017 Dec 27;13(12):e1006788. doi: 10.1371/journal.ppat.1006788 (PMC5770078; doi:10.1371/journal.ppat.1006788)

S1 Fig.

A

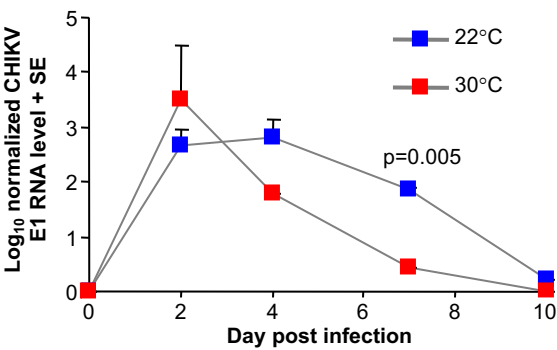

B

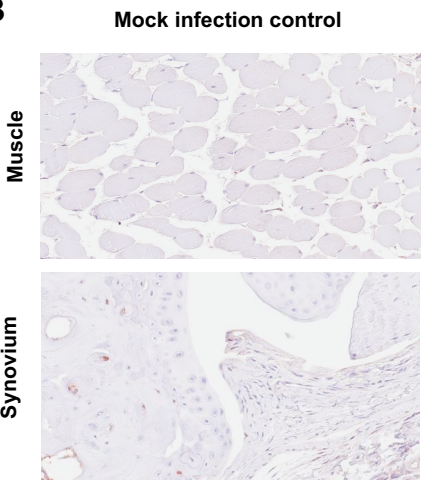

Supplement: S1 Fig — (A) qRT PCR for CHIKV E1 of feet of mice held at 22°C and 30°C. C57BL/6 mice were infected s.c. in the feet as for Fig 1 and at the indicated times feet were harvested (n = 3 feet from 3 mice per time point and temperature) and CHIKV E1 RNA levels determined by qRT PCR as in Fig 1B. Statistics by t test. (B) Immunohistochemistry as in Fig 1F on mock infected mouse tissues. (PDF) [file ppat.1006788.s001.pdf]

S2 Fig.

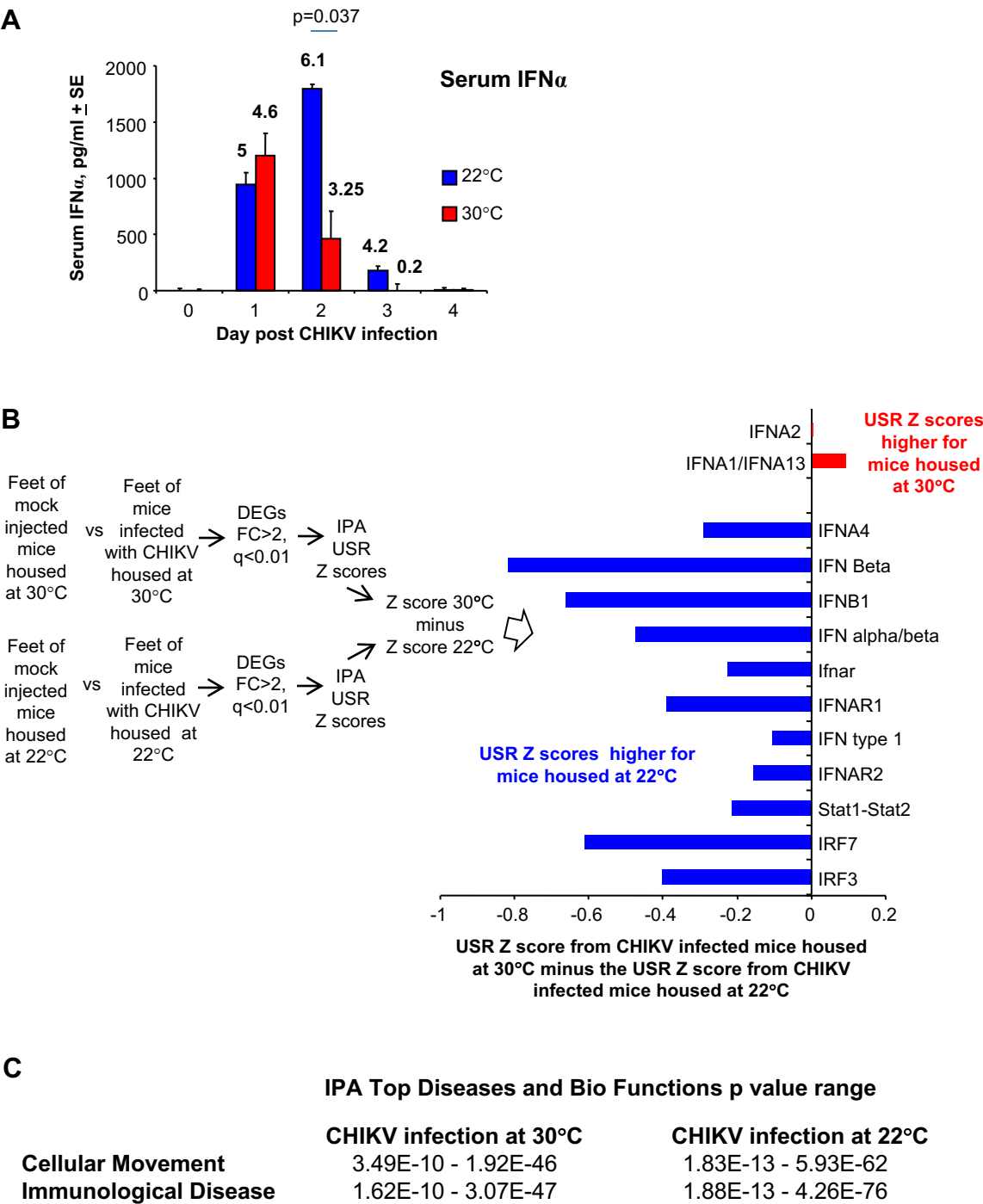

Supplement: S2 Fig — (A) Mice were housed at 22°C or 30°C (n = 5/6 per group) and were infected s.c. in the feet with CHIKV as in Fig 1. Serum IFNα levels were then determined at the indicated times post infection. The numbers above the bars represent the mean viremias in log10CCID50/ml. On days 2 and 3 the mean viremias were 3–4 logs higher in mice housed at 22°C. (B) RNA-Seq analysis of day 2 feet from mice infected with CHIKV was performed as described [27], with mice housed at 30°C or 22°C. DEGs for both temperatures were determined relative to mock infected mice housed at the same temperature. DEGs (q<0.01, fold change >2) were analyzed using the Upstream Regulator (USR) feature of Ingenuity Pathway analysis (IPA). As expected, given the higher viremia in mice housed at 22°C (see above), most USR pathways associated with the type I IFN response had higher Z scores for mice housed at 22°C than for mice housed at 30°C. However, despite the difference in viral loads, USR Z scores for well annotated IFNαs that are involved in the secondary amplification loop were actually higher for mice housed at 30°C than for mice housed at 22°C. (C) Using the same DEG lists as in B and the “Top Diseases and Bio Functions” feature of IPA, “Immunological Disease” and “Cellular Movement” showed higher significance (lower p value ranges) for mice housed at 22°C than for mice housed at 30°C. This is consistent with the H&E data shown in Fig 1. (PDF) [file ppat.1006788.s002.pdf]

S3 Fig.

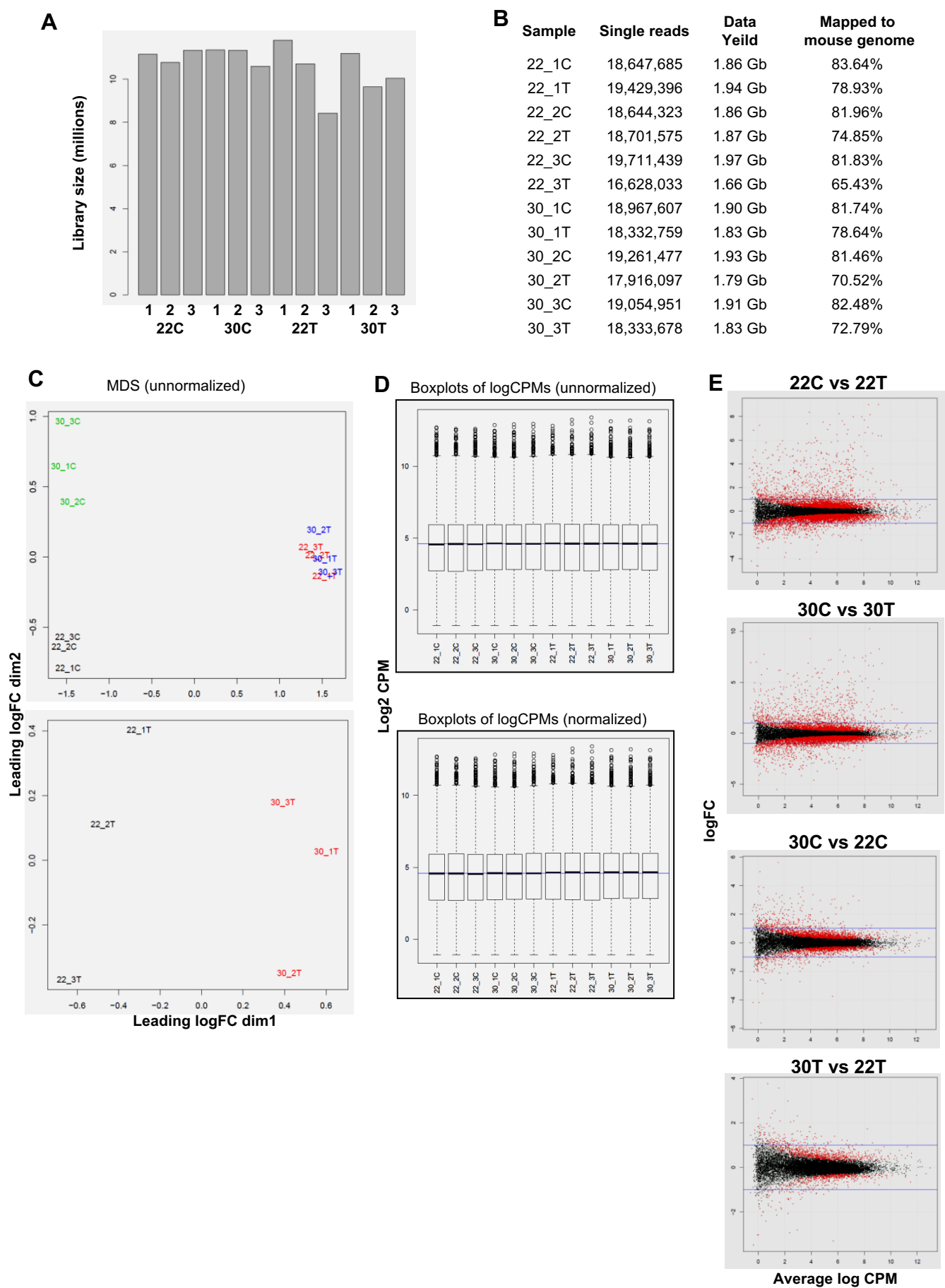

Supplement: S3 Fig — (A) Library sizes. (T refers to Treatment with poly(I:C)/jetPEI and C to no treatment Controls). (B) Read numbers, data yield and percentage of reads mapping to the mouse genome. The per base sequence quality was high, with >84% of bases above Q30 for all 12 samples. (C) MDS clustering for all samples, and for 30T vs 22T samples. (D) Boxplots showing the distribution of expression values for all samples before and after normalization. (E) Smear plots: log-fold-change plotted against the log counts per million. Genes showing significant differences (FDR<0.05) are in red. Blue lines represent log2 fold change of 1 and -1 (i.e. fold change of 2 and -2). (PDF) [file ppat.1006788.s003.pdf]

S4 Fig.

Up regulated genes

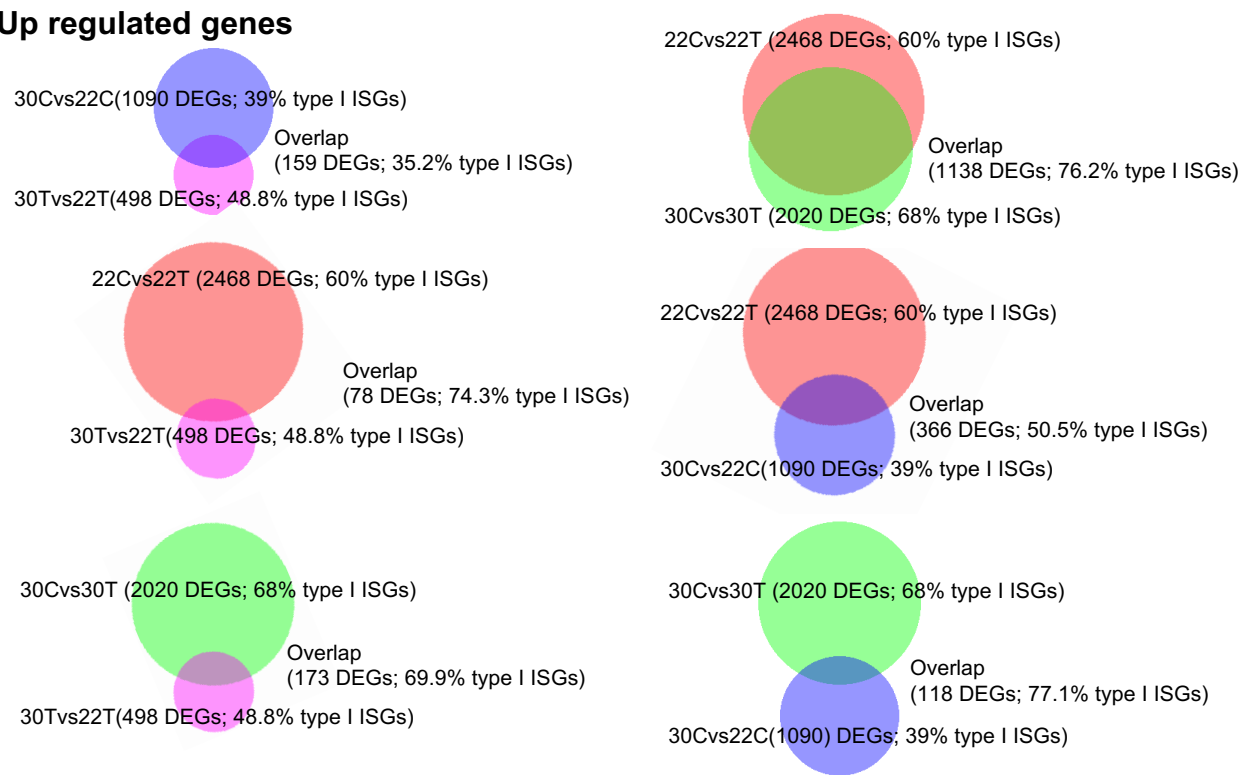

Down regulated genes

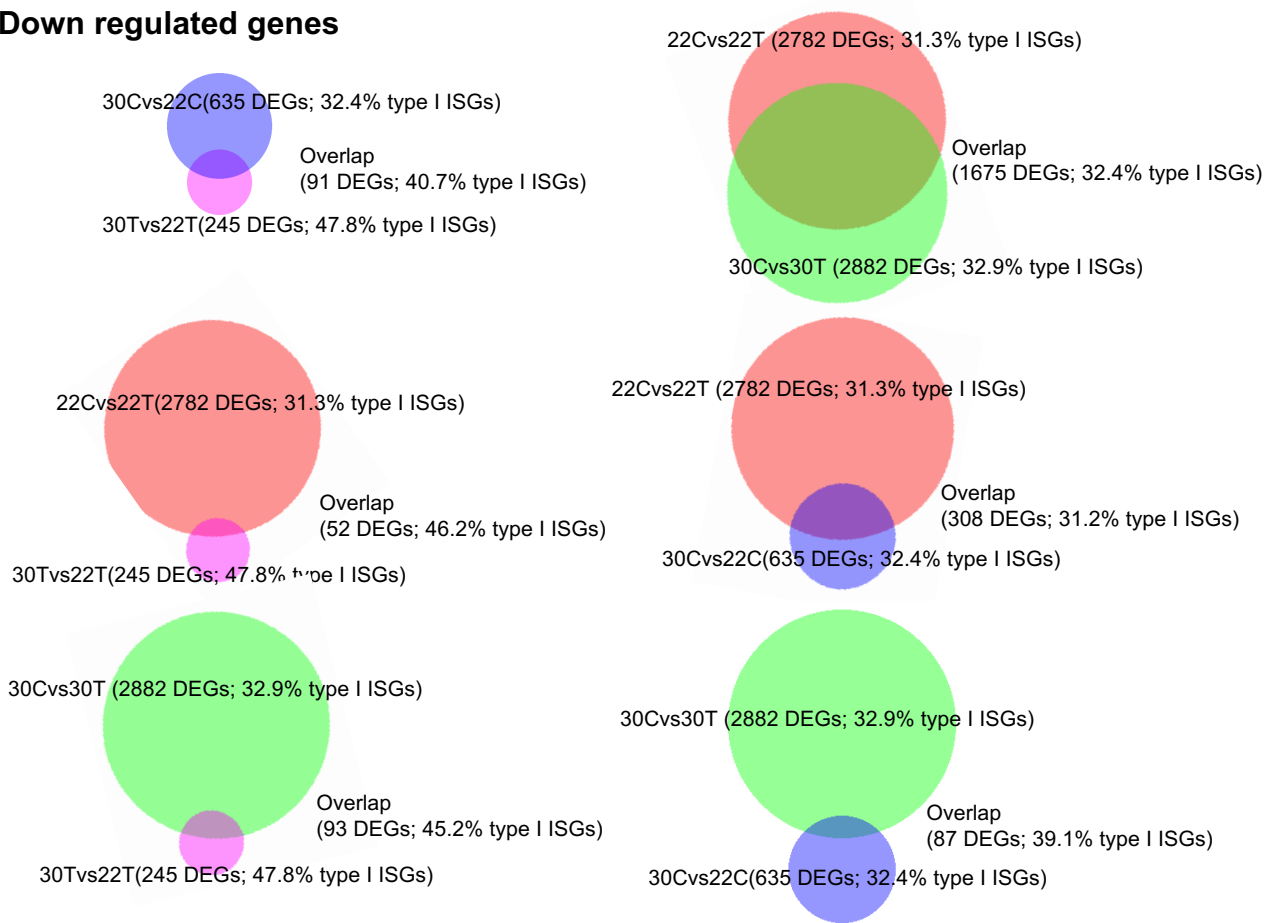

Supplement: S4 Fig — Overlaps of DEGs (FDR<0.01, CPM>1 in at least 3 samples) for 30Cvs22C, 30Tvs22T, 22Cvs22T and 30Cvs30T are show for up and down regulated genes. Type I interferon stimulated genes (ISGs) were determined by Interferome (v2.0), selecting type I interferon and default settings (All) for all other search conditions. (PDF) [file ppat.1006788.s004.pdf]

S6 Fig.

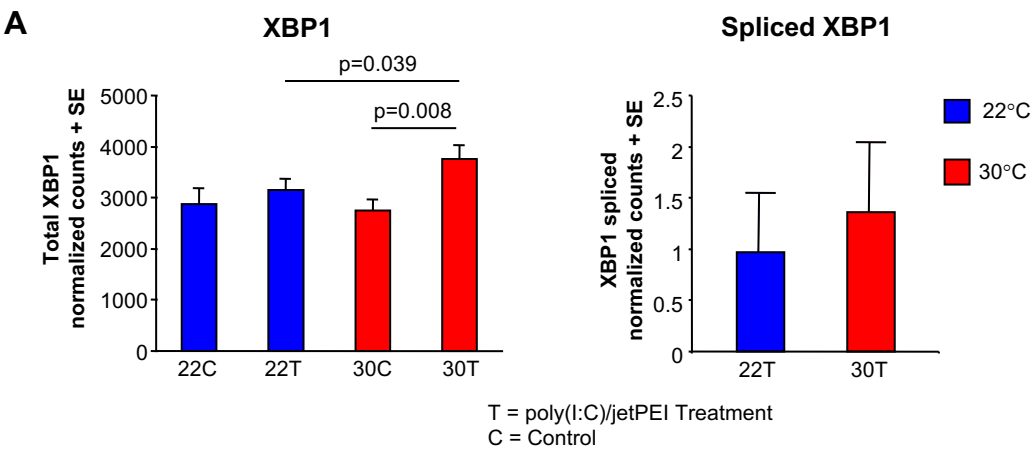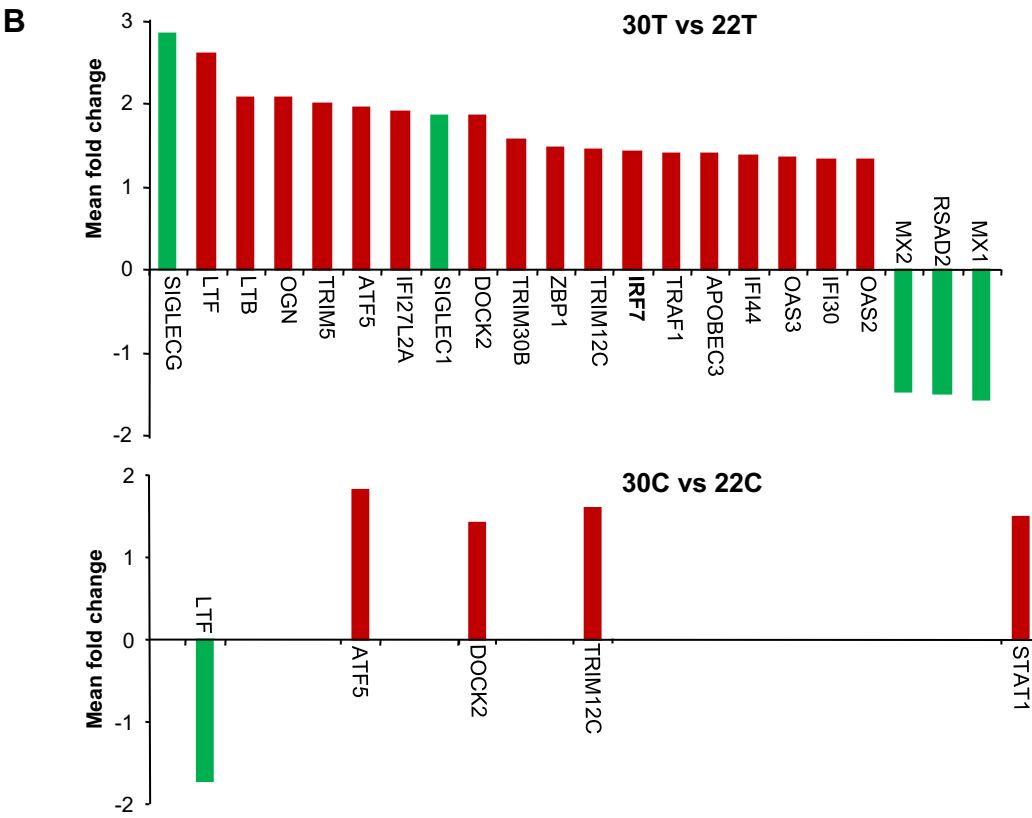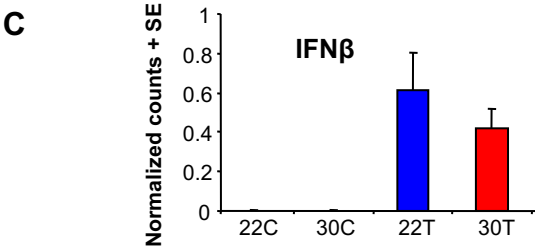

Supplement: S6 Fig — (A) XBP1 mRNA levels after poly(I:C)/jetPEI injection of mice housed at 22°C or 30°C derived from the RNA-Seq data (Statistics by t tests). Spliced XBP1 levels were determined using the Sashimi feature of the Integrative Genome Viewer (IGV) v2.3.34. (B) Using the DEG lists for 30T vs 22T (top graph) and 30C vs 22C (bottom graph) (q<0.01) the mean fold change (no log transformation applied) for selected genes where the change in expression would be associated with promotion of anti-viral activities (red) or where the change in expression would be associated with reduction of anti-viral activities (green). IRF7 is bolded. (C) IFNβ mRNA expression. Normalized counts for IFNβ mRNA for each condition; n = 3 for each. Poly(I:C)/JetPEI induced IFNβ mRNA expression at both temperatures (i.e. 22C vs 22T and 30C vs 30T), but levels were not significantly different for 30T and 22T. (PDF) [file ppat.1006788.s006.pdf]

S7 Fig.

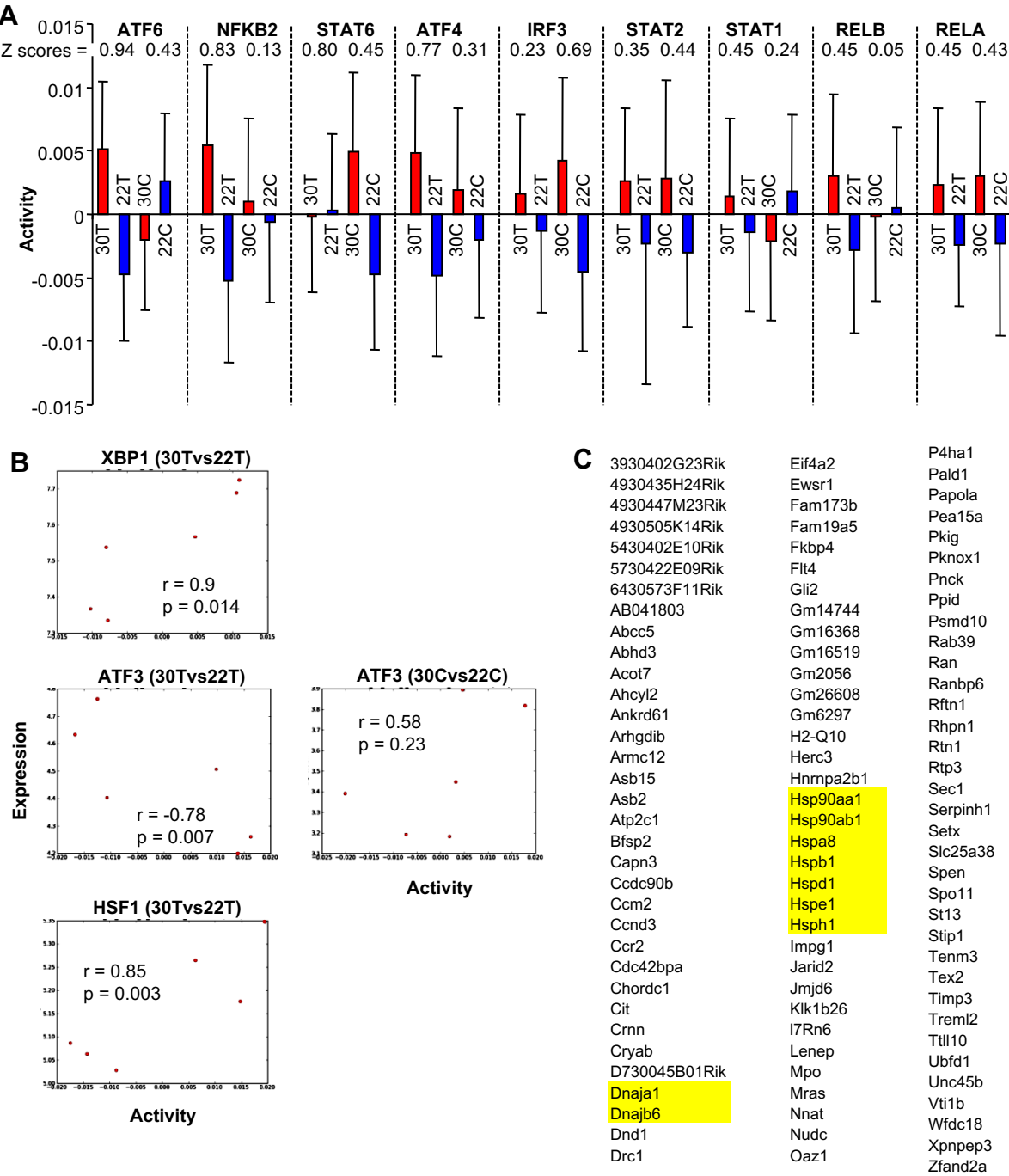

Supplement: S7 Fig — (A) Activity and Z scores for promoter site activities for selected transcription factors associated with the type I interferon response. Analyses as for Fig 6C. The activity of the indicated transcription factors nearly always tended to be higher at 30°C, although only XBP1 and ATF3 (see Fig 6C) reached Z scores >2 and non-overlapping error bars, conditions for clear significance recommend by ISMARA protocols. A unique IRF7 site is not available in ISMARA. (B) Expression/Activity correlations. ISMARA Expression/Activity correlations showing Pearson’s correlation coefficients (r) and p values for transcription factor sites shown in Fig 6C. (C) HSF1 target genes (identified by ISMARA) for HSF1 for 30T vs 22T, with heat shock proteins highlighted in yellow. (PDF) [file ppat.1006788.s007.pdf]

S8 Fig.

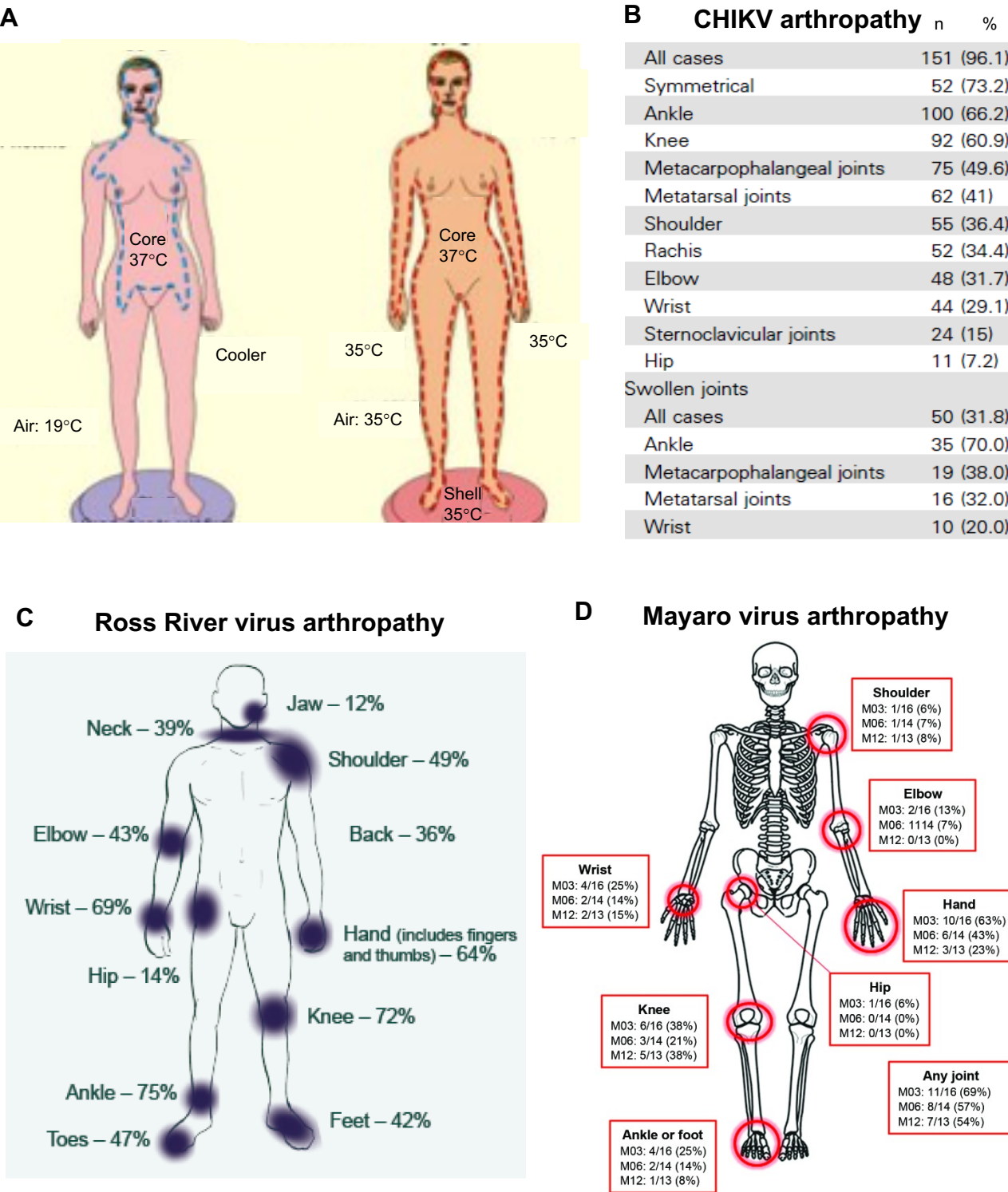

Supplement: S8 Fig — (A) Limb and body temperatures in humans (adapted from New Human Physiology 2nd Edition. Chapter 21: Thermo-Regulation, Temperature and Radiation. Eds. Paulev PE, Zubieta-Calleja G. Copenhagen, Denmark). (B) The number of patients (n) and the percentage of CHIKV patients (%) with arthropathy in the indicated joints (n = 71 patients) (taken from Borgherini et al., 2007 [26]). (C) The percentage of RRV patients with arthritic disease in the indicated joints (for example; 69% of RRV patients report pain and/or swelling in their wrist joints) (produced by Environmental Health Directorate, Dept. Health, WA, Australia, 2006). (D) Joints affected in Mayaro virus patients (taken from Halsey et al., 2013 [25]). M03, M06 and M12 represent 3, 6, and 12 months follow-up. The numerator represents the number of patients reporting arthropathy in the indicated joints, the denominator the number of patients present at the indicated follow-up time. The percentage is numerator/denominator x100. (PDF) [file ppat.1006788.s008.pdf]

**A**

### 30C vs 22C

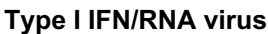

**UPR**

**B**

## 30T vs 22T

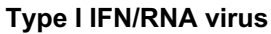

**UPR**

Supplement: S9 Fig — Using the commercial GeneGo MetaCore software (http://portal.genego.com), genes associated with (i) the unfolded protein response (UPR) (blue) and (ii) Type I IFN response together with Response to RNA virus (green) were uploaded (GO module). Networks were constructed and merged (Build network module) and expression data from (A) 30Cvs22C or (B) 30Tvs22T applied as a filter to the merged networks. The two networks intersect at a number of nodes, and even without treatment feet of mice housed at 30°C show up-regulation (red circles) of genes associated with anti-viral responses. (PDF) [file ppat.1006788.s009.pdf]

S10 Fig.

A

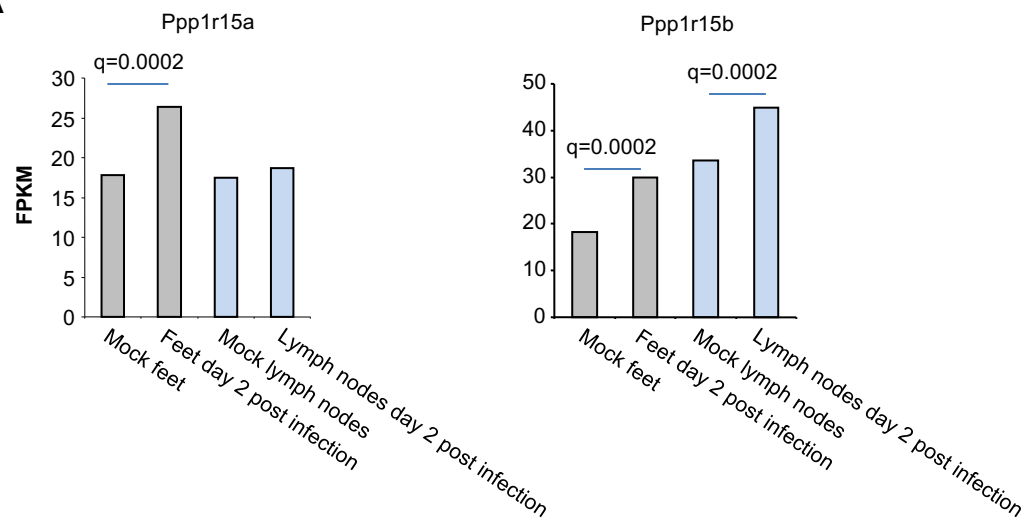

B

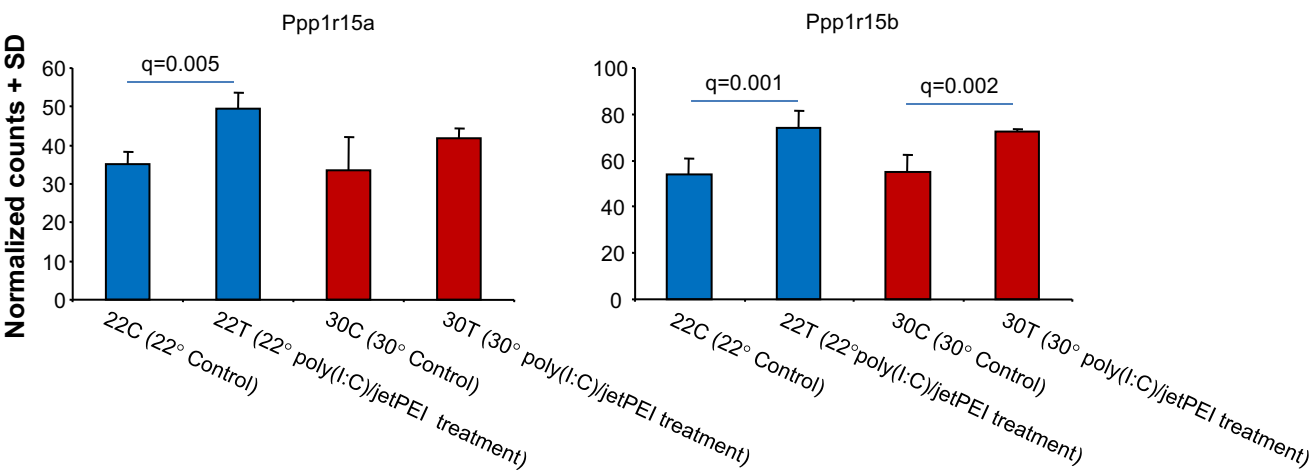

Supplement: S10 Fig — A major consequence of the UPR and responses to dsRNA (via PKR [110]) is translation inhibition, which appears to promote anti-viral responses via reduced translation of inhibitors (e.g. A20, SHIP-1, IкBα) [94]. GADD34-mediated relief of translational repression (via eIF2α dephosphorylation and/or stress granule dissolution) is required for IFNβ protein synthesis [94,95]. (A) GADD34 mRNA is up-regulated in feet and lymph nodes 2 days post CHIKV infection (using the RNA-Seq data set described in Wilson et al 2017); a result consistent with [95]. (B) Although GADD34 mRNA was also up-regulated in feet at 12 h after poly(I:C)/JetPEI injection, there were no significant differences for mice housed at 30°C. (PDF) [file ppat.1006788.s010.pdf]

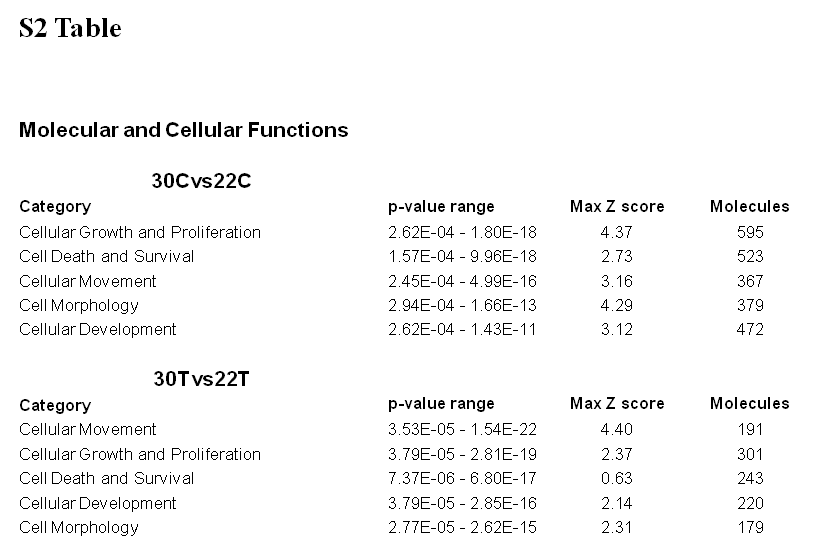

Supplement: S2 Table — Genes associated with cell growth, survival, movement, morphology and development are up-regulated in feet of mice held at 30°C both in the absence of treatment (30C vs 22C) and after poly(I:C)/jetPEI treatment (30T vs 22T). IPA “Molecular and Cellular Functions” analysis (direct only) for all DEGs (up and down regulated) was undertaken for 30Cvs22C and 30Tvs22T (FDR<0.01, CPM >1 in at least 3 samples). (“Molecular and Cellular Functions” is a sub-category of the “Diseases and Functions” feature, which returns a number of “Diseases or Functions Annotations” for each of the listed categories; thus providing a p value range). (DOCX) [file ppat.1006788.s012.docx]
