# Supplementary material for: Lower temperatures reduce type I interferon activity and promote alphaviral arthritis
Source: PLoS Pathog. 2017 Dec 27;13(12):e1006788. doi: 10.1371/journal.ppat.1006788 (PMC5770078; doi:10.1371/journal.ppat.1006788)
Supplement: S5 Fig — (A) Gene set enrichment analyses (GSEA 3.0, Broad Institute) comparing DEGs (fold change >2, q <0.01) 12 hours after poly(I:C)/jetPEI injection (as in S4 Fig) and 2 days after CHIKV infection (as in S2B Fig). (B) ISGs significantly induced (fold change >2, q <0.01) on day 2 post CHIKV infection [27] show considerable overlap with ISGs significantly induced 12 hours after poly(I:C)/jetPEI injection. ISGs were identified via Interferome (selecting type I interferon and default settings ‘All’ for all other search conditions). (PDF) [file ppat.1006788.s005.pdf]

S5 Fig.

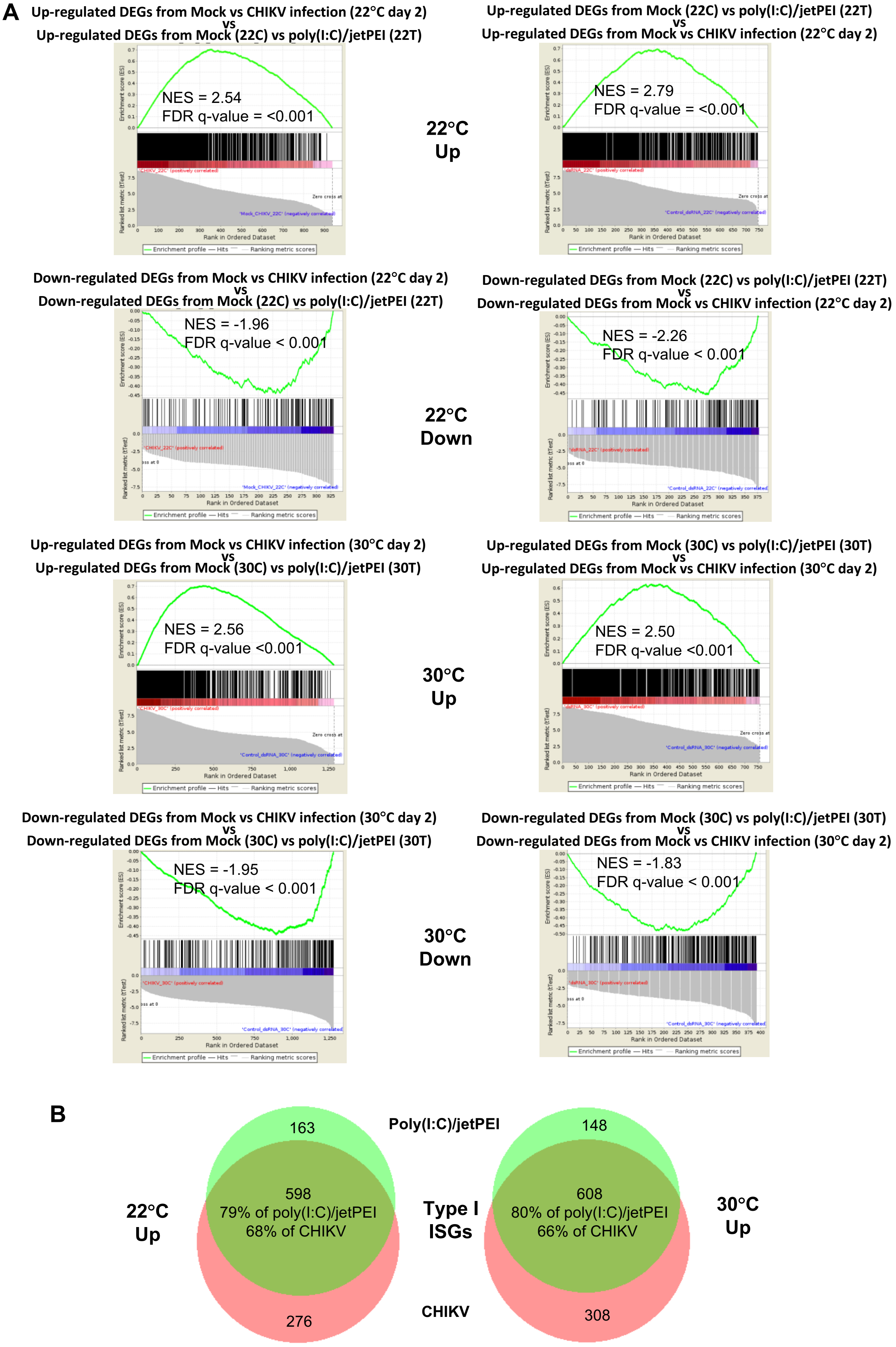

A
Up-regulated DEGs from Mock vs CHIKV infection (22°C day 2)
vs
Up-regulated DEGs from Mock (22C) vs poly(I:C)/jetPEI (22T)
NES = 2.54
FDR q-value = <0.001
Up-regulated DEGs from Mock (22C) vs poly(I:C)/jetPEI (22T)
vs
Up-regulated DEGs from Mock vs CHIKV infection (22°C day 2)
NES = 2.79
FDR q-value = <0.001
22°C
Up
Down-regulated DEGs from Mock vs CHIKV infection (22°C day 2)
vs
Down-regulated DEGs from Mock (22C) vs poly(I:C)/jetPEI (22T)
NES = -1.96
FDR q-value < 0.001
Down-regulated DEGs from Mock (22C) vs poly(I:C)/jetPEI (22T)
vs
Down-regulated DEGs from Mock vs CHIKV infection (22°C day 2)
NES = -2.26
FDR q-value < 0.001
22°C
Down
Up-regulated DEGs from Mock vs CHIKV infection (30°C day 2)
vs
Up-regulated DEGs from Mock (30C) vs poly(I:C)/jetPEI (30T)
NES = 2.56
FDR q-value <0.001
Up-regulated DEGs from Mock (30C) vs poly(I:C)/jetPEI (30T)
vs
Up-regulated DEGs from Mock vs CHIKV infection (30°C day 2)
NES = 2.50
FDR q-value <0.001
30°C
Up
Down-regulated DEGs from Mock vs CHIKV infection (30°C day 2)
vs
Down-regulated DEGs from Mock (30C) vs poly(I:C)/jetPEI (30T)
NES = -1.95
FDR q-value < 0.001
Down-regulated DEGs from Mock (30C) vs poly(I:C)/jetPEI (30T)
vs
Down-regulated DEGs from Mock vs CHIKV infection (30°C day 2)
NES = -1.83
FDR q-value < 0.001
30°C
Down
Enrichment score (ES)
Ranked list metric (tTest)
Rank in Ordered Dataset
Enrichment profile — Hits — Ranking metric scores
B
Poly(I:C)/jetPEI
163
598
79% of poly(I:C)/jetPEI
68% of CHIKV
276
22°C
Up
Type I
ISGs
CHIKV
148
608
80% of poly(I:C)/jetPEI
66% of CHIKV
308
30°C
Up
